# Biomarkers of Response to Low-Dose Aspirin in Familial Adenomatous Polyposis Patients

**DOI:** 10.3390/cancers15092457

**Published:** 2023-04-25

**Authors:** Angel Lanas, Stefania Tacconelli, Annalisa Contursi, Elena Piazuelo, Annalisa Bruno, Maurizio Ronci, Simone Marcone, Melania Dovizio, Federico Sopeña, Lorenza Falcone, Cristina Milillo, Matteo Mucci, Patrizia Ballerini, Paola Patrignani

**Affiliations:** 1 University Hospital LB, Aragon Health Research Institute (IISAragon), CIBERehd, University of Zaragoza, 50009 Zaragoza, Spain; fsopena@unizar.es; 2 Center for Advanced Studies and Technology (CAST), “G. d’Annunzio” University, 66100 Chieti, Italyannalisa.contursi@unich.it (A.C.); a.bruno@unich.it (A.B.); m.dovizio@unich.it (M.D.); lorenza.falcone@unich.it (L.F.); cristina.milillo@unich.it (C.M.); matteo.mucci@unich.it (M.M.); patrizia.ballerini@unich.it (P.B.); 3 Department of Neuroscience, Imaging and Clinical Science, “G. d’Annunzio” University, 66100 Chieti, Italy; 4 Instituto Aragonés de Ciencias de la Salud (IACS), 50009 Zaragoza, Spain; epiazor@unizar.es; 5 Department of Medical, Oral and Biotechnological Sciences, “G. d’Annunzio” University, 66100 Chieti, Italy; maurizio.ronci@unich.it; 6 Trinity Translational Medicine Institute, Trinity College Dublin, D02 PN40 Dublin, Ireland; marcones@tcd.ie; 7 Department of Innovative Technologies in Medicine and Dentistry, “G. d’Annunzio” University, 66100 Chieti, Italy

**Keywords:** platelets, colorectal adenomas, cyclooxygenases, COX acetylation, thromboxane, prostaglandin E_2_, familial adenomatous polyposis

## Abstract

**Simple Summary:**

Aspirin has been studied for a long time in familial adenomatous polyposis (FAP), but the data on the prevention of colorectal adenoma formation and recurrence remain controversial. We conducted a biomarker-based clinical study in eight FAP patients treated with low-dose Aspirin to clarify the drug’s mechanism of action. Aspirin appropriately acetylated platelet cyclooxygenase (COX)-1 but left persistently high systemic thromboxane(TX)A_2_ and prostaglandin (PG)E_2_ biosynthesis (assessed by measuring their primary urinary metabolites, i.e., 11-dehydro-TXB_2_ and PGEM) associated with the incomplete acetylation of COX-1 in colorectal mucosa and adenomas. Proteomics of colorectal adenomas of FAP patients showed that the vimentin upregulation and hemoglobin subunit beta downregulation distinguished the FAP patients in two groups with high vs. low residual 11-dehydro-TXB_2_ levels, possibly identifying Aspirin nonresponders and responders, respectively. Novel chemotherapeutic strategies in FAP may involve drugs that affect TXA_2_ and PGE_2_ signaling in the colorectum with receptor antagonists, and clinical studies should be performed to verify the drugs’ impact.

**Abstract:**

Background: The results of Aspirin prevention of colorectal adenomas in patients with familial adenomatous polyposis (FAP) are controversial. Methods: We conducted a biomarker-based clinical study in eight FAP patients treated with enteric-coated low-dose Aspirin (100 mg daily for three months) to explore whether the drug targets mainly platelet cyclooxygenase (COX)-1 or affects extraplatelet cellular sources expressing COX-isozymes and/or off-target effects in colorectal adenomas. Results: In FAP patients, low-dose Aspirin-acetylated platelet COX-1 at Serine529 (>70%) was associated with an almost complete inhibition of platelet thromboxane (TX) B_2_ generation ex vivo (serum TXB_2_). However, enhanced residual urinary 11-dehydro-TXB_2_ and urinary PGEM, primary metabolites of TXA_2_ and prostaglandin (PG)E_2_, respectively, were detected in association with incomplete acetylation of COX-1 in normal colorectal biopsies and adenomas. Proteomics of adenomas showed that Aspirin significantly modulated only eight proteins. The upregulation of vimentin and downregulation of HBB (hemoglobin subunit beta) distinguished two groups with high vs. low residual 11-dehydro-TXB_2_ levels, possibly identifying the nonresponders and responders to Aspirin. Conclusions: Although low-dose Aspirin appropriately inhibited the platelet, persistently high systemic TXA_2_ and PGE_2_ biosynthesis were found, plausibly for a marginal inhibitory effect on prostanoid biosynthesis in the colorectum. Novel chemotherapeutic strategies in FAP can involve blocking the effects of TXA_2_ and PGE_2_ signaling with receptor antagonists.

## 1. Introduction

Familial Adenomatous Polyposis (FAP) is inherited in an autosomal-dominant manner and results from germline mutations in the adenomatous polyposis (*APC*) gene, a tumor-suppressor gene that controls the Wnt/β-catenin signaling pathway. *APC* mutations are responsible for 70–90% of FAP cases [1]. *MUTYH* is the second FAP-related gene, and it is involved with the base-excision repair of DNA damaged by oxidative stress. *MUTYH* mutations are inherited in an autosomal-recessive manner and account for 10–20% of classical FAP cases [2]. FAP manifests equally in both sexes and accounts for less than 1% of colorectal cancer (CRC) cases [3]. In FAP, more than 100 precancerous polyps initiate to grow in childhood and adolescence, which can transform into CRC during the fourth and fifth decades of life if not treated [4]. The safest preventive strategy is surgical resection; however, following colectomy, patients are at a 25% risk of developing cancer in the retained rectum after 20 years [5,6]. Thus, these patients undergo regular endoscopic controls to individuate and treat loco-regional relapse.

Aspirin (acetylsalicylic acid, ASA) is a nonsteroidal anti-inflammatory drug (NSAID) that has been evaluated to prevent colorectal adenomas in patients with FAP [7,8]. The adenoma/Carcinoma Prevention Programme 1 (CAPP1) trial, a randomized, placebo-controlled trial of daily Aspirin (600 mg: 300 mg BID, a medium dose) and/or resistant starch (30 g), showed no significant reduction in polyp count (the primary endpoint) or size of the largest polyps (secondary endpoint) with either intervention. However, a significant reduction in polyp size among patients treated with Aspirin for more than one year was detected [7]. Recently, Ishikawa et al. [8] performed a double-blind, placebo-controlled trial in 104 patients with FAP assessing the effects of low-dose ASA (100 mg/day) and mesalazine (2 g/day) on the recurrence of colorectal polyps (J-FAPP Study IV) in 11 centers in Japan. The drug suppressed the recurrence of colorectal polyps larger than 5 mm (primary endpoint) in FAP patients [8].

Aspirin acts by acetylating (cyclooxygenase) COX-1 and COX-2 at Serine529 and Serine516, respectively, thus leading to the irreversible inactivation of the COX activity of the enzymes and inhibiting the biosynthesis of prostanoids [9,10]. The administration of Aspirin at low doses (such as 100 mg daily) preferentially targets platelet COX-1 [11]. It profoundly inhibits the biosynthesis of the potent proaggregatory and vasoconstrictor mediator thromboxane (TX)A_2_. For this effect, the drug is recommended for the secondary prevention of cardiovascular disease [12].

The mechanism of action of Aspirin as an antitumor agent in FAP is still under debate. Thus, we aimed to conduct a biomarker-based clinical study in 8 FAP patients treated chronically with enteric-coated (EC) low-dose Aspirin (100 mg daily for three months) to explore whether it inhibits mainly platelet COX-1 or whether it can affect colorectal COX-1 and COX-2 or causes off-target effects. To realize these aims, we assessed the impact of the drug on: (i) the extent of COX-1 acetylation at Serine529 (primary endpoint) and COX-2 acetylation at Serine516 in colorectal adenomas, normal mucosa, and platelets; (ii) the systemic biosynthesis of prostanoids; (iii) the expression of enzymes and receptors of prostanoid pathways in platelets and intestinal tissue; and (iv) proteomics of FAP patients’ adenomas.

## 2. Materials and Methods

### 2.1. Clinical Study, Participant Characteristics, and Sample Collections

**Figure 1:**
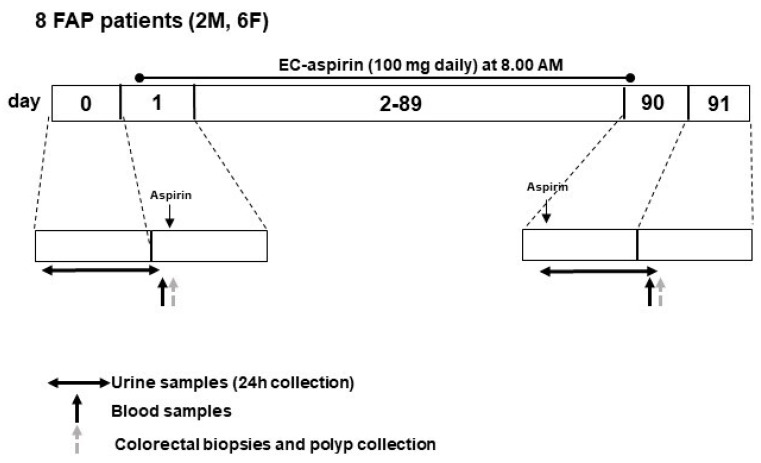
Flow chart of the clinical study protocol.

Eleven patients with an established (genetic and phenotypic) diagnosis of FAP were enrolled in the “Lozano Blesa” Clinic Hospital of Zaragoza (Spain), and signed, informed consent for the study was obtained. Enrolled patients could withdraw their consent at any time during the study. This study complied with the Declaration of Helsinki and was performed according to the Regional Ethics Committee of Aragon’s (CEICA) approval (EudraCT:2013-004269-15). The study design and inclusion and exclusion criteria for patient enrolment are reported in the Supplementary Materials. In particular, the patients should not present cardiovascular risk factors and not be smokers. Three FAP patients were excluded because they presented hypercholesteremia. Thus, 8 FAP patients were selected for treatment with enteric-coated Aspirin (EC-Aspirin 100 mg/daily, Adiro, Bayer) for three consecutive months (Figure 1).

Their demographic, clinical, and genetic features are reported in Table 1. To consenting patients, at baseline and after Aspirin administration, the following samples were collected: (i) urine samples (24-hour collections), (ii) blood samples, and (iii) intestinal biopsies, including polyps and normal tissue of the rectum and left and right colon obtained by colonoscopy. The procedures for sample collection and storage are reported in Supplementary Material. In 8 healthy subjects matched for age and sex (Table 1), 24-hour urine samples were collected at baseline to evaluate the systemic biosynthesis of prostanoids by measuring the urinary levels of major enzymatic metabolites [13,14].

### 2.2. Assessment of Biomarkers

In 8 FAP patients, at baseline and 3 months after the Aspirin treatment, platelet COX-1 activity ex vivo was studied by evaluating the production of TXB_2_ in whole blood allowed to clot for 1 h at 37 °C (serum TXB_2_), as previously described [13,14,15]. The effects on systemic biosynthesis of the main prostanoids, i.e., TXA_2_, prostaglandin (PG)E_2_, PGI_2_ (prostacyclin), and PGD_2_, were assessed by measuring the urinary levels of their primary urinary enzymatic metabolites, i.e., TXM (11-dehydro-TXB_2_ and the minor metabolite 2,3-dinor-TXB_2_), PGEM (7-hydroxy-5,11-diketotetranorprostane-1,16-dioic acid), PGIM (2,3-dinor-6-keto-PGF_1α_), and PGDM (11,15-dioxo-9α-hydroxy-2,3,4,5-tetranorprostan-1,20-dioic acid), by liquid chromatography-mass spectrometry (LC-MS/MS) as previously reported [13,14]. We assessed the capacity of low-dose Aspirin to acetylate COX-1 at Serine529 in washed platelets by LC-MS/MS as previously described [13,14,16]. In colorectal adenomas and normal tissue biopsies of the rectum and left and right colon, we assessed the % acetylation of COX-1 at Serine529 and COX-2 at Serine516 by LC-MS/MS as previously described [13,14,17]. In platelets, colorectal adenomas, and the normal tissue of the left and right colon and rectum, the protein levels of COX-1 and COX-2, TXA_2_ synthase (TXS), and TXA_2_ receptors (TP) were assessed by Western blot as previously described [14] and reported in detail in Supplementary Material. In 4 patients (randomly selected), colorectal adenomas and the normal tissue of the left and right colon and rectum were collected to assess the expression of genes involved in the PGE_2_ biosynthetic pathway *PTGS1* (COX-1), *PTGS2* (COX-2), *PTGES* (Microsomal prostaglandin E synthase-1, mPGES-1), *PTGES3* (Cytosolic prostaglandin E_2_ synthase, cPGES), and *HPGD* (15-Hydroxyprostaglandin dehydrogenase, 15-PGDH) by quantitative PCR (qPCR), as reported below. In six patients (randomly selected), colorectal adenomas were collected to perform proteomics analyses by LC-MS/MS, as described below.

### 2.3. Animal Study

*Apc^Min/+^* mice and their wild-type (*WT*) littermates on the C57BL/6J background were purchased from the Jackson Lab (Bar Harbor, ME, USA) and bred according to The Jackson Laboratory Handbook on Genetically Standardized Mice. The animal experiments were performed under the European Communities Council (EEC) Directive of 22 September 2010 (2010/63/EU) and the National Ethical Committee (authorization n. 956/2016-PR). *Apc^Min/+^* mice (*n* = 4, 50% F) and their *WT* littermates C57BL/6J (*n* = 4, 50% F) were sacrificed at 120 days of age when *Apc^Min/+^* mice develop spontaneous intestinal adenomas [18]. At sacrifice, intestinal adenomas from *Apc^Min/+^* mice and norma3l intestinal mucosa from *WT* mice were collected as previously reported [18] for gene expression analysis of *Ptgs1* (COX-1), *Ptgs2* (COX-2), *Tbxas1* (TXS), *Tbxa2r* (TXA_2_ receptor, TP), *Vim* (vimentin), *Il6* (interleukin 6), *Ptges* (mPGES-1), *Ptges3* (cPGES), *Hpgd* (15-PGDH), *Pla2g4a* (cytosolic phospholipase A_2,_ cPLA2), *Il1b* (interleukin 1 beta), *Twist1* (twist basic helix-loop-helix transcription factor 1), *Cdh1* (E-cadherin), *Ptger2* (prostaglandin E receptor 2, EP2), *Ptger4* (prostaglandin E receptor 4, EP4), *Myc* (c-Myc), and *Gapdh* (glyceraldehyde-3-phosphate dehydrogenase) and were assessed by qPCR, as previously described [18] and briefly reported below.

### 2.4. Assessment of Gene Expression by qPCR

Colorectal adenomas and normal tissue samples of FAP patients, adenomas of *Apc^Min/+^* mice, and normal mucosa of *WT* mice samples were homogenized with TissueRuptor (Qiagen, MD, USA); and total RNA was extracted using Pure link RNA Mini kit (Life Technologies, Carlsbad, CA, USA) according to the manufacturer’s protocols. Total RNA (2 μg) was treated with DNAse kit (Fermentas, St. Leon-Rot, Germany) and reverse-transcribed into cDNA using Iscript cDNA Synthesis Kit (Bio-Rad, Milan, Italy) according to the manufacturer’s protocols. One hundred nanograms of cDNA were used for the reaction mixture.

For the assessment of gene expression in colorectal samples of FAP patients, the amplification of *PTGS1*, *PTGS2*, *PTGES*, *HPGD*, *PTGES3*, *GAPDH*, *VIM*, and *HBB* was performed using TaqMan gene expression assays [Hs00377726, Hs00153133, Hs01115610, Hs00960586, Hs00832847, Hs99999905, Hs00185584, and Hs00758889, respectively (Applied Biosystems, Foster City, CA, USA)] according to the manufacturer’s instructions using a 7900HT Real-Time PCR system (Applied Biosystems, Foster City, CA, USA).

For the assessment of gene expression in intestinal samples of mice, the amplification of *Ptgs1*, *Ptgs2*, *Tbxas1*, *Tbxa2r*, *Vim*, *Il6*, *Ptges*, *Ptges3*, *Hpgd*, *Pla2g4a*, *Il1b*, *Twist1*, *Cdh1*, *Ptger2*, *Ptger4*, *Myc*, and *Gadph* was performed using TaqMan gene expression assays (mm00477214, mm00478374, mm00495553, mm00436917, mm01333430, mm00446190, mm00452105, mm00727367, mm00515121, mm00447040, mm00434228, mm00442036, mm01247357, mm00436051, mm00436053, mm01192721, and mm99999915, respectively) (Applied Biosystems) according to the manufacturer’s instructions using a 7900HT Real-Time PCR system (Applied Biosystems). Gene expression assays were performed by relative quantification with comparative cycle threshold (Ct) using ABI Prism, SDS 2.4 software (Applied Biosystems).

### 2.5. Proteomics of FAP Colorectal Adenomas

#### 2.5.1. Protein Extraction and Filter-Aided Sample Preparation

Samples were lysed as previously described [14], and proteins were quantified through the Bradford assay. Fifty μg of proteins were loaded onto a Nanosep 10-kDa-cutoff filter (Pall Corporation—Ann Arbor, MI, USA) and digested according to a protocol adapted from Dister et al. [19]. Briefly, the proteins were washed twice with 200 μL urea buffer (8 M urea, 100 mM tris pH 8.5 in milliQ water) to remove the detergents present in the lysis buffer and subsequently reduced and alkylated by adding 100 μL of 8 mM dithiothreitol solution in urea buffer and 100 μL 50 mM iodoacetamide solution in urea buffer. After urea buffer exchange with 50 mM ammonium bicarbonate, the trypsin digestion solution (Sigma, St. Louis, MO, USA) was added to a ratio of 1:50 (enzyme: substrate). After overnight incubation at 37 °C, the resulting peptides were collected by centrifugation, acidified with 10% trifluoroacetic acid (Sigma, St. Louis, MO, USA), and stored at −20 °C until analysis.

#### 2.5.2. LC-MS/MS Label-Free Shotgun Proteomics

Each digested protein sample was analyzed in technical duplicate by LC-MS/MS using a Proxeon EASY-nLCII (Thermo Fisher Scientific, Milan, Italy) chromatographic system coupled to a Maxis HD UHR-TOF (Bruker Daltonics GmbH, Bremen, Germany) mass spectrometer as previously reported [20].

#### 2.5.3. Raw Data Processing and Quantitative Analysis

Raw mass spectrometry data were processed with PEAKS^®^ Studio 7.5 software [21] using the ‘correct precursor only’ option. Spectra were searched against the UniProtKB/Swiss-Prot human database to which a list of common contaminants was appended (20,441 entries), and the false discovery rate (FDR) was set to 0.1% at the peptide-spectrum matches (PSM) level. Database search options were set as follows: fixed cysteine carbamidomethylation (ΔMass: 57.02), variable methionine oxidation (ΔMass: 15.99), and glutamine and asparagine deamidation (ΔMass: 0.98). Nonspecific cleavage was allowed to 1 end of the peptides, with a maximum of 2 missed cleavages and Trypsin enzyme specificity. Error mass tolerances for precursors and fragments were set at 10 ppm and 0.05 Da, respectively. Differentially expressed proteins were detected through the label-free quantification (LFQ) tool of PEAKS Studio with the following parameters: Mass Error Tolerance: 10.0 ppm; Retention Time Shift Tolerance: 1.0 min; and FDR Threshold: 0.5%. The significance threshold at the protein level was set to ≥20 −10 lgP with fold change ≥2.0.

STRINGv11 was used to generate protein–protein interaction networks. The Database for Annotation, Visualization, and Integrated Discovery (DAVID v2022q2) was used to understand the biological meaning behind the lists of genes. The identified proteins were also subjected to enrichment and network analysis through the free web tool ProteoMill (proteomill.com ver.11.0).

### 2.6. Statistical Analysis

All values were reported as mean ± SD unless otherwise stated. Statistical analysis among groups was determined by a two-tailed Student’s *t*-test and one-way or two-way ANOVA, and multiple comparisons were performed using Tukey’s test, using GraphPad Prism Software (version 9.00 for Mac; GraphPad, San Diego, CA, USA). Values of *p* < 0.05 were considered statistically significant. The primary hypothesis was that administering EC-Aspirin would cause maximal acetylation of platelet COX-1 (70%) and partial acetylation of COX-1 (50%) expressed in colorectal tissue. Based on a previous study performed on healthy subjects, we calculated an intersubject coefficient of variation (CV) of platelet AceCOX-1 of 10% by Aspirin [13]. Thus, a sample size of 8 individuals had a 90% power to detect a difference between means of COX-1 acetylation of platelets and colorectal tissue of 17.47 or more with a significance level (alpha) of 0.05 (two-tailed).

## 3. Results

### 3.1. Enhanced Systemic Biosynthesis of Prostanoids in FAP

**Figure 2:**
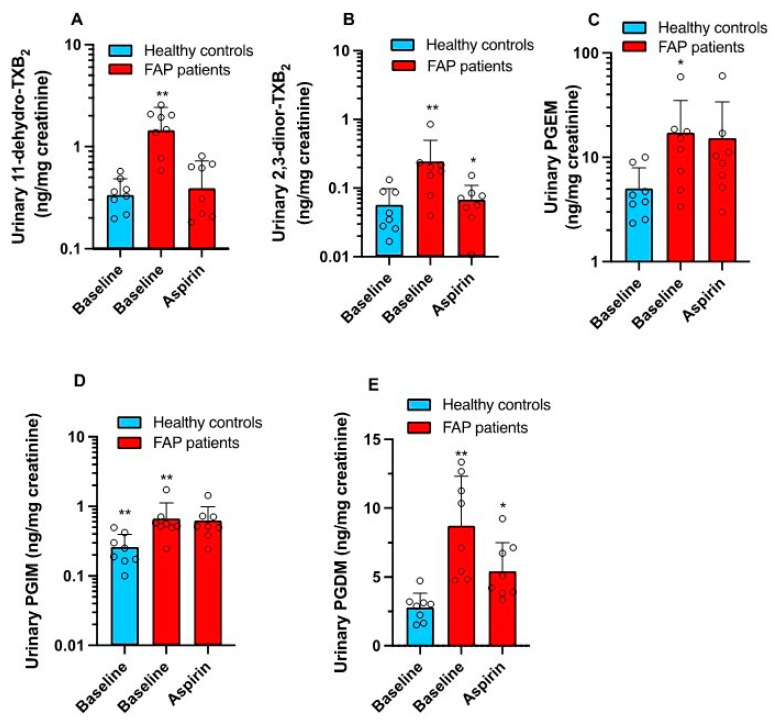
Systemic biosynthesis of TXA_2_, PGE_2_, PGI_2,_ and PGD_2_. In 8 FAP patients and 8 healthy controls matched for age and sex, the urinary levels of TXM [11-dehydro-TXB_2_ (**A**) and 2,3-dinor-TXB_2_ (**B**)], PGEM (**C**), PGIM (**D**), and PGDM (**E**) were assessed at baseline by LC-MS/MS. In FAP patients, 24-hour urine samples were collected after 3 months of treatment with 100 mg daily of enteric-coated Aspirin to assess the same urinary metabolites (**A**–**E**). Urinary metabolite levels (ng/mg creatinine) were transformed to logarithms if they were not normally distributed, and all values are shown as scatter dot plots with mean + SD and analyzed by one-way ANOVA followed by Tukey’s multiple comparisons test. (**A**) ** *p* < 0.01 vs. baseline healthy controls and FAP aspirin; (**B**) ** *p* < 0.01 vs. baseline healthy controls, * *p* < 0.05 vs. FAP baseline; (**C**) * *p* < 0.05 vs. baseline healthy controls; (**D**) ** *p* < 0.01 vs. FAP patients (baseline) and FAP patients (Aspirin); (**E**) ** *p* < 0.01 vs. baseline healthy controls, * *p* < 0.05 vs. FAP patients (baseline).

At baseline, FAP patients presented higher systemic biosynthesis of TXA_2_, PGE_2_, PGI_2_, and PGD_2_ than the healthy controls, matched for age and sex, as shown by the significantly higher urinary levels of their primary enzymatic metabolites TXM (11-dehydro-TXB_2_ and 2,3-dinor-TXB_2_) (Figure 2A and Figure 2B, respectively), PGEM (Figure 2C), PGIM (Figure 2D), and PGDM (Figure 2E), respectively.

### 3.2. Effects of Low-Dose Aspirin on the Acetylation of COX-Isozymes

**Figure 3:**
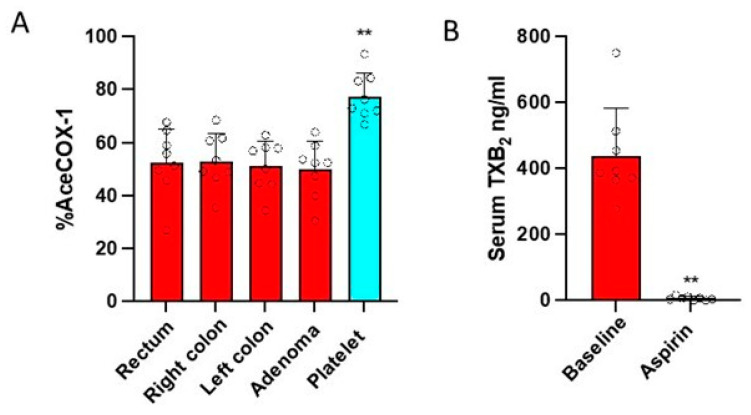
(**A**) Effects of treatment with low-dose Aspirin in FAP patients on the % acetylation of COX-1 in isolated platelets and colorectal biopsies. The extent of acetylation of COX-1 (%AceCOX-1) was assessed in isolated platelets, colorectal adenomas, and biopsies of the colon (left and right) and rectum by LC-MS/MS. All values are shown as scatter dot plots with mean + SD (*n* = 8), ** *p* < 0.01 vs. all other conditions using one-way ANOVA and Tukey’s posthoc test. (**B**) Effects of low-dose Aspirin on platelet COX-1 activity of FAP patients. Serum was obtained by allowing whole blood samples to clot for 1 h at 37 °C, and TXB_2_ was assessed by a validated immunoassay. ** *p* < 0.01 vs. Aspirin using two-tailed Student’s *t*-test.

In FAP patients treated with low-dose EC-Aspirin (100 mg daily for 3 months), 24 h after the last administration, the acetylation of platelet COX-1 at Serine529 was significantly higher than in colorectal adenomas and normal tissue collected on the left and right colon and rectum (Figure 3A). We could not detect significant levels of COX-2 and its acetylated form at Serine516 in any colorectal sample by LC-MS/MS.

### 3.3. Effects of Low-Dose Aspirin on Prostanoid Biosynthesis

The administration of low-dose Aspirin to FAP patients caused virtually complete inhibition of serum TXB_2_ (98.7 ± 1.0%; a marker of platelet COX-1 activity) (Figure 3B) 24 h after the last dose of the drug.

Aspirin comparably reduced the urinary levels of 11-dehydro-TXB_2_ and 2,3-dinor-TXB_2_ vs. baseline (predrug) by approximately 70%, i.e., the fraction derived from platelet COX-1 (Figure 2A,B). Residual levels of these metabolites measured after Aspirin were not significantly different from baseline values found in the healthy subjects (Figure 2A,B). Aspirin did not significantly affect enhanced urinary PGEM or PGIM (Figure 2C,D), considered mainly of COX-2 origin. Aspirin significantly reduced enhanced systemic PGD_2_ biosynthesis vs. predrug in FAP, but residual values were still higher than the baseline PGDM levels of healthy subjects (Figure 2E).

### 3.4. Effects of Low-Dose Aspirin on the Expression of Enzymes and Receptors of Prostanoid Pathways in Platelets and Intestinal Tissue

**Figure 4:**
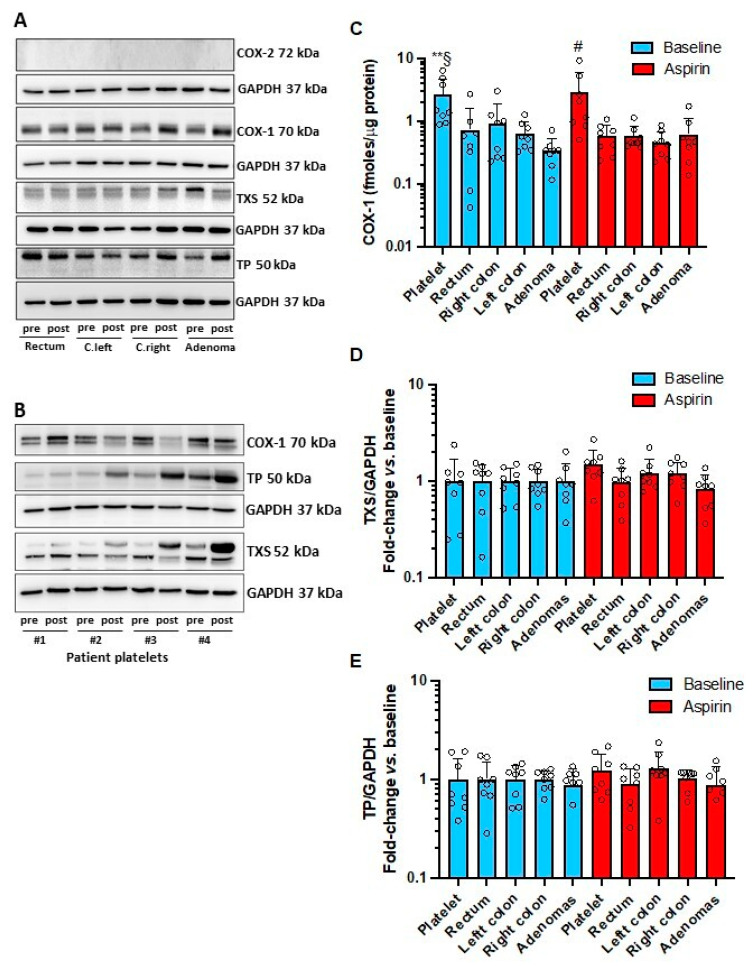
Protein levels of COX-1, COX-2, TXA_2_ synthase (TXS), and TXA_2_ receptors (TP) in colorectal biopsies and platelets of FAP patients before and after Aspirin treatment. (**A**) Representative Western blots of COX-2, COX-1, TXS, TP, and GAPDH in tissue biopsies [rectum, biopsies of left colon (c. left) and right colon (c. right) and colorectal adenomas] before (pre) and after Aspirin treatment (post). (**B**) Representative Western blots of isolated platelets (performed in 4 FAP patients, i.e., #1–4) for COX-1, TP, TXS, and GAPDH, before (pre) and after Aspirin treatment (post); for TXS, a nonspecific band with higher molecular weight (MW) is shown. (**C**) COX-1 levels were quantified by LC-MS/MS in the tissue biopsies (colorectal adenomas, biopsies of left and right colon and rectum) and isolated platelets at baseline and after Aspirin treatment. (**D**) Fold change vs. baseline of TXS protein expression by densitometric analysis of Western blot bands corrected for the levels of GAPDH as a loading control. (**E**) Fold change vs. baseline of TP receptor protein expression by densitometric analysis of Western blot bands corrected for the levels of GAPDH as a loading control. All data are reported as scatter dot plots with mean + SD, *n* = 7, 8; data that did not pass the normality test were log-transformed before the analysis by two-way ANOVA followed by Tukey’s multiple comparisons test. (**C**) Baseline: § *p* < 0.05 vs. left colon and right colon, ** *p* < 0.01 vs. rectum and adenoma, Aspirin: # *p* < 0.01 vs. rectum, right colon, left colon, and adenoma. Original blot are shown in Supplementary Figures S1 and S2.

COX-1 protein was detected in normal colorectal biopsies, adenomas, and platelets by Western blot and LC-MS/MS (Figure 4A–C, Figures S1 and S2). Platelet COX-1 was higher than in colorectum tissue (Figure 4C); low-dose Aspirin did not affect COX-1 protein levels (Figure 4A–C). COX-2 was not detected in any sample analyzed (Figure 4A). TXA_2_ synthase (TXS) and TXA_2_ receptor (TP) were detected in platelets and colorectal biopsies to a comparable extent, and Aspirin did not significantly affect them (Figure 4A,B,D,E, Figures S1 and S2). However, an increase of the two proteins was detected in the platelets of some Aspirin-treated patients vs. baseline (Figure 4B).

**Figure 5:**
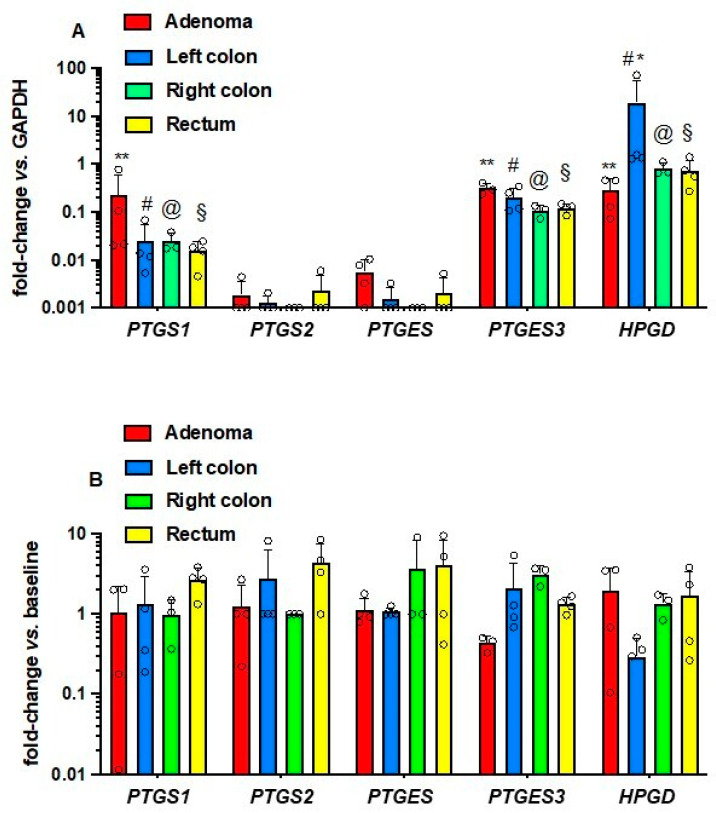
Gene expression of COX-1, COX-2, mPGES-1, cPGES, and 15-PGDH in colorectal adenomas, the normal tissue of the left and right colon and rectum of FAP patients at baseline (**A**) and after Aspirin (**B**). The mRNA levels of *PTGS1* (protein name: COX-1), *PTGS2* (protein name COX-2), *PTGES* (protein name: prostaglandin E synthase, also called mPGES-1), *PTGES3* (protein name: Prostaglandin E synthase 3, also called cPGES), *HPGD* (protein name: 15-PGDH), and *GAPDH* were assessed by qPCR in intestinal adenomas and apparently normal tissues of the left and right colon and rectum at baseline (before-Aspirin) (**A**) and after-Aspirin treatment (**B**). In panel A, gene expression is reported as fold change vs. *GAPDH*. In panel B, the data are shown as a fold change from baseline values (before Aspirin). All data are reported as scatter dot plots with mean + SD, *n* = 3–4. Since data did not pass the normality test, they were transformed into logarithms. All data were analyzed by two-way ANOVA followed by Tukey’s multiple comparisons test. (**A**) **, #, @, § *p* < 0.01 vs. COX-2 and mPGES-1; * *p* < 0.05 vs. adenoma, and right colon and rectum.

The expression of genes involved in the PGE_2_ biosynthetic pathway was assessed by qPCR since the antibodies for Western blot were not sensitive enough and did not give reproducible bands in our hands. As shown in Figure 5A, *PTGS2* (COX-2) and *PTGES* (mPGES-1) were expressed at low levels. In contrast, *PTGS1* (COX-1) and *PTGES3* (cPGES) were expressed at higher levels suggesting an important contribution to colorectal tissue PGE_2_ biosynthesis; their expression levels were comparable in adenomas and normal colorectal mucosa. The expression of *HPGD* encoding 15-PGDH, involved in the degradation of PGE_2_ to a less active metabolite [22], was not significantly different among colorectal adenomas and the right colon and rectum biopsies. In contrast, in the left colon, 15-PGDH expression was significantly higher (Figure 5A) than in the other intestinal tissue samples. Low-dose Aspirin did not significantly affect the expression levels of these genes in colorectal normal tissues and adenomas (Figure 5B).

### 3.5. Enhanced Gene Expression of Inflammatory Pathways in the Intestinal Adenomas of Apc^Min/+^ vs. Normal Tissue of WT Mice

**Figure 6:**
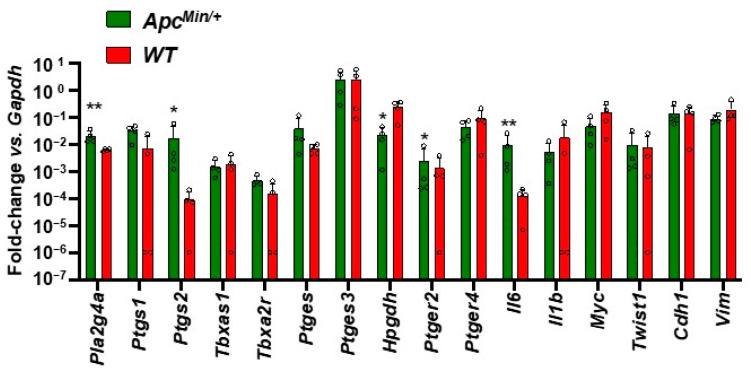
Targeted gene expression in intestinal adenomas of *Apc^Min/+^* mice and normal intestinal mucosa of *WT* mice. *Ptgs1* (protein name: prostaglandin-endoperoxide synthase 1, COX-1), *Ptgs2* (protein name: prostaglandin-endoperoxide synthase 2, COX-2), *Tbxas1* (protein name: thromboxane A synthase 1, TXAS), *Tbxa2r* (protein name: thromboxane A_2_ receptor), *Vim* (protein name: Vimentin), *Il6* (protein name: interleukin 6), *Ptges* (protein name: prostaglandin E synthase, mPGES-1), *Ptges3* (protein name: prostaglandin E synthase 3, cPGES), *Hpgd* (protein name: hydroxyprostaglandin dehydrogenase 15 (NAD), 15-PGDH), *Pla2g4a* (protein name: cytosolic phospholipase A2), *Il1b* (protein name: interleukin 1 beta), *Twist1* (protein name: twist basic helix-loop-helix transcription factor 1), *Cdh1* (protein name: cadherin 1, E-cad), *Ptger2* (protein name: prostaglandin E receptor 2, EP2), *Ptger4* (protein name: prostaglandin E receptor 4, EP4), *Myc* (protein name: myelocytomatosis oncogene), and *Gapdh* (protein name: glyceraldehyde-3-phosphate dehydrogenase) were assessed by qPCR in intestinal adenomas of *Apc^Min/+^* mice (*n* = 4) and normal intestinal mucosa of *WT* mice (*n* = 4). Gene expression is reported as fold change vs. *Gapdh*. All data are reported as scatter dot plots with mean + SD, *n* = 4. Since data did not pass the normality test, they were transformed into logarithms. All data were analyzed by multiple unpaired *t*-test. ** *p* < 0.01 vs. *WT*; * *p* < 0.05 vs. *WT*.

Whether altered gene expression in intestinal adenomas could contribute to the enhanced systemic biosynthesis of prostanoid biosynthesis detected in FAP vs. healthy subjects was verified in Apc^Min/+^(an animal model of FAP [23,24]) vs. WT mice. We analyzed targeted gene expression profiling by qPCR of adenomas of Apc^Min/+^ mice and normal intestinal tissue of WT mice (Figure 6). We found that intestinal adenomas of Apc^Min/+^ mice manifest higher relative expression of genes encoding cPLA2, COX-2, and IL-6 than normal intestinal tissue of WT mice. Also, enhanced expression of the protumorigenic PGE_2_ receptor EP2 occurred. In contrast, the expression of the tumor suppressor 15-PGDH was significantly reduced in adenomas of Apc^Min/+^ mice vs. the normal intestinal tissue of WT mice. The expression of genes involved in epithelial-mesenchymal transition (EMT) was not changed significantly (Figure 6).

### 3.6. Proteomics of FAP Patients’ Adenomas and Aspirin Effects

**Figure 7:**
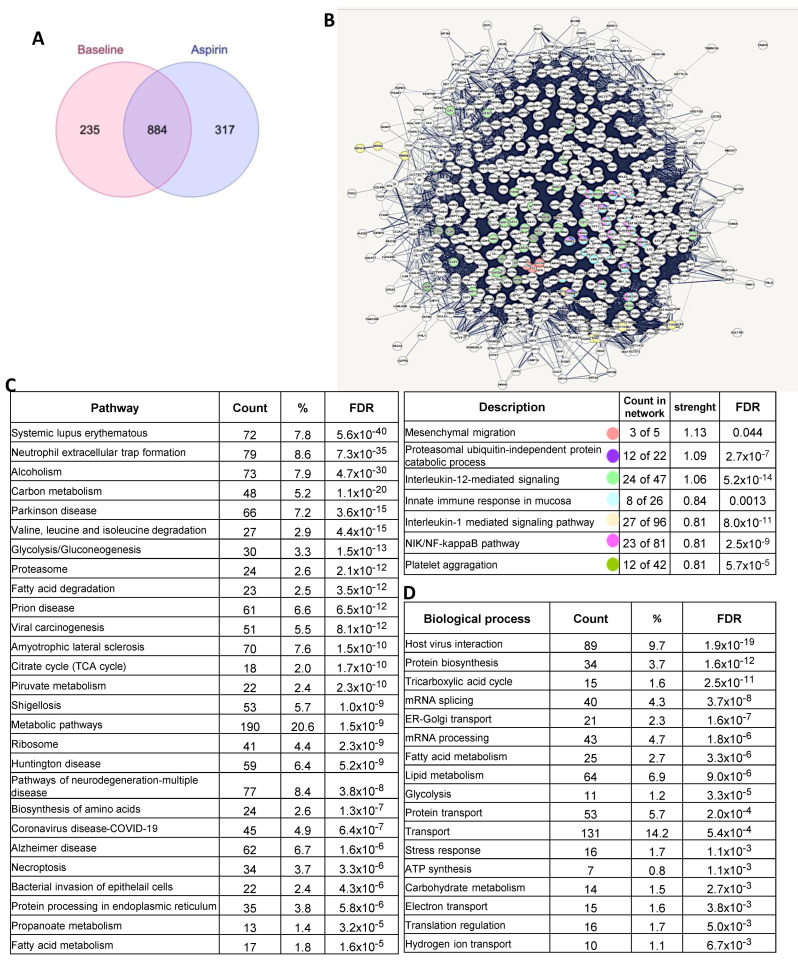
Proteomics of FAP patient adenomas collected at baseline and after chronic treatment with low-dose Aspirin. (**A**) Using the multiple list comparator (a free online tool), identified proteins at baseline and after Aspirin treatment were compared, and the Venn diagram is shown. (**B**) The shared proteins were selected for network and pathway analysis (using STRING v11); the proteins interaction network of the 884 identified proteins (shown as nodes in the network) are reported, and proteins associated with relevant biological processes (Gene Ontology) are highlighted with colors. (**C**) Shared proteins were submitted to DAVID for Gene Ontology analysis, and the results for KEGG pathway analysis are reported. (**D**) Biological processes were obtained using DAVID Bioinformatics Resources.

The finding that low-dose Aspirin can acetylate COX-1 expressed in colorectal adenomas suggests that the drug targets the colorectal tissue of FAP patients. Thus, we compared the proteomics of adenomas collected at predrug (baseline) and after chronic treatment with low-dose Aspirin in six FAP patients. We identified 1436 proteins (Table S1), and 884 proteins were common at baseline and after the aspirin treatment (Figure 7A). The interaction network of these proteins is reported in Figure 7B, and the proteins associated with mesenchymal migration and inflammation networks are highlighted with colors. The most highly enriched biological processes (by FDR) using DAVID Bioinformatics Resources are related to host–virus interaction, lipid metabolism, protein biosynthesis, and mRNA processing, and the top-ranked KEGG pathways involved among them were inflammatory-driven diseases and viral carcinogenesis and bacterial invasion epithelial cells (Figure 7C,D).

**Figure 8:**
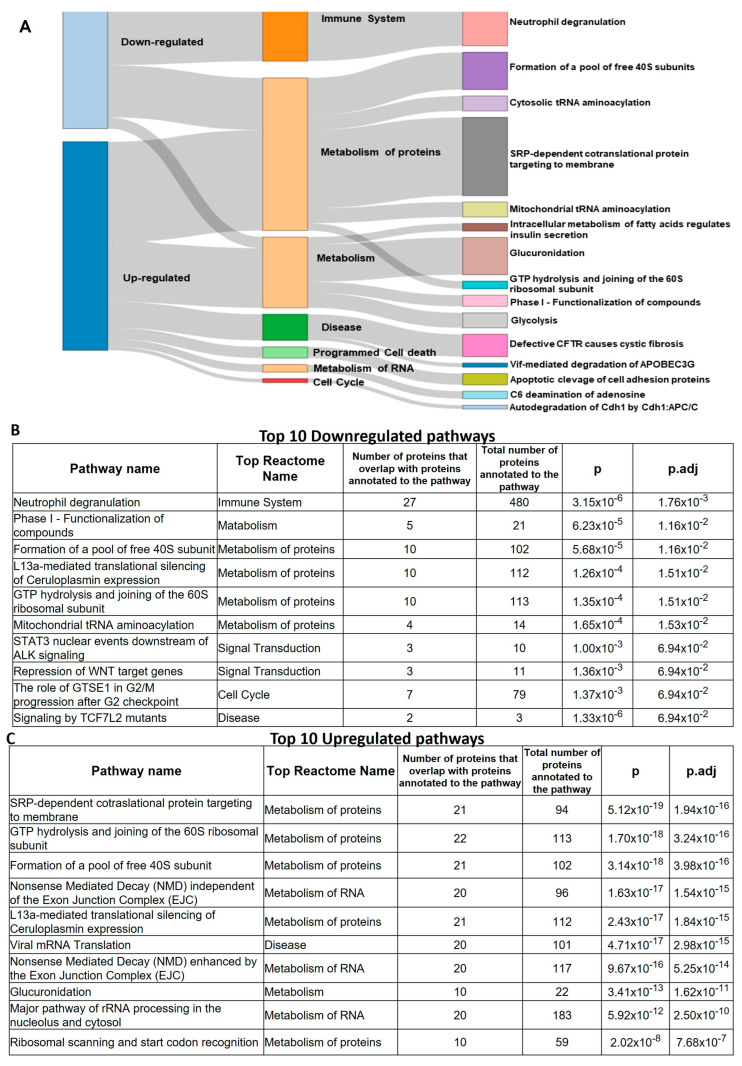
Proteomics of FAP patient adenomas collected at baseline and after chronic treatment with low-dose Aspirin. Proteins identified exclusively at baseline T1 (baseline) (235 proteins) or T2 (after Aspirin treatment) (317 proteins) were considered upregulated or downregulated, respectively. They were subjected to enrichment and network analysis through the free web tool ProteoMill. (**A**) The Sankey diagram shows the up- and downregulated pathways resulting from the enrichment analysis: neutrophil degranulation resulted in the most significantly enriched downregulated pathway, and SRP-dependent cotranslational protein targeting to membrane the most significantly enriched upregulated pathway. (**B**,**C**) The top 10 most enriched up- and downregulated pathways are shown.

Two hundred thirty-five and 317 proteins uniquely expressed at predrug- and after-Aspirin treatments, respectively (Figure 7A), were considered upregulated or downregulated, respectively, and were subjected to enrichment and network analysis through the free web tool ProteoMill (https://proteomill.com/, accessed on 5 March 2023) (Figure 8A–C). The Sankey diagram shows the up- and downregulated pathways from the enrichment analysis (Figure 8A). Neutrophil degranulation resulted in the most significantly enriched downregulated pathway (Figure 8B), and signal recognition particle (SRP)-dependent cotranslational protein targeting to the membrane was the most significantly enriched upregulated pathway (Figure 8C).

**Figure 9:**
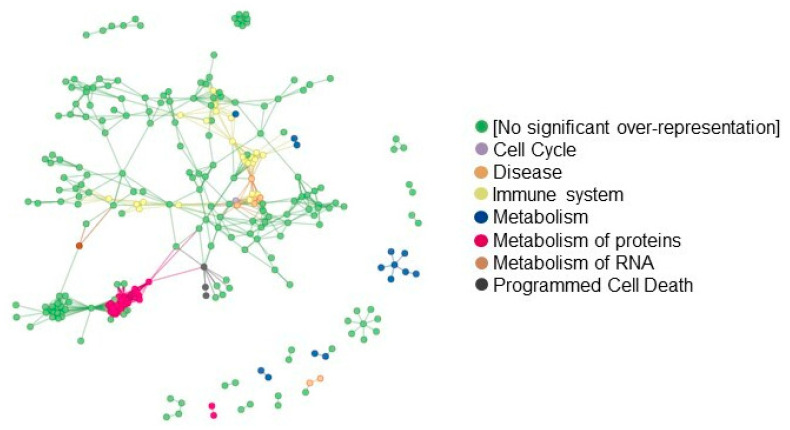
Interaction network of the 552 adenoma proteins identified exclusively at baseline (T1) and after low-dose Aspirin (T2). The corresponding colors are coded to the Reactome Names. Interaction confidence is set to 7/10, which means moderately good evidence supporting the interactions being presented.

The protein interaction network of the 552 up- and downregulated proteins through ProteoMill interrogating the REACTOME pathway database (with an FDR < 0.01) is reported in Figure 9. Interaction confidence is set to 7/10, which means moderately good amounts of evidence in support of the interactions being presented.

**Figure 10:**
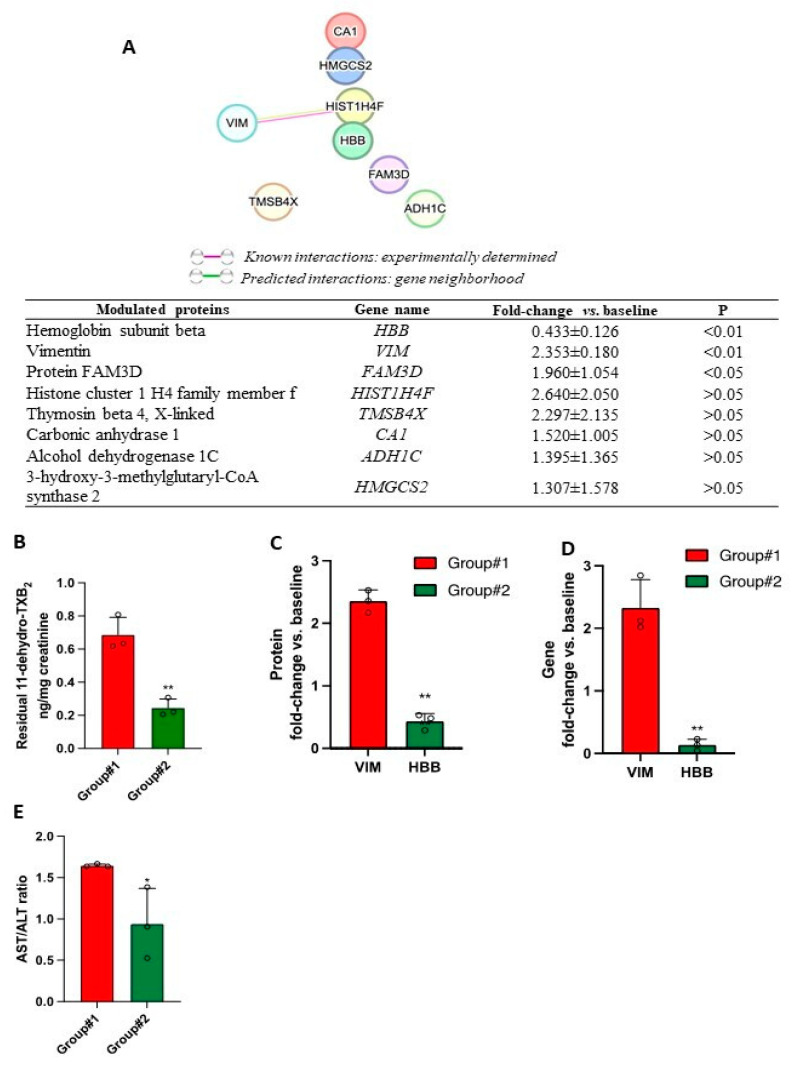
Adenoma-modulated proteins by Aspirin treatment. (**A**) Gene and protein names of the significantly modulated proteins by Aspirin treatment in FAP patients. (**B**,**C**) Residual 11-dehydro-TXB_2_ in Aspirin-treated FAP patients with upregulated *VIM* (group #1) and downregulated *HBB* (group #2) values are reported as scatter dot plots with mean + SD, *n* = 3 each group. (**D**) Fold change of the expression of *VIM* and *HBB* in adenoma of FAP patients treated with Aspirin vs. predrug by qPCR values are reported as scatter dot plots with mean + SD, *n* = 3. (**E**) Serum AST/ALT, respectively, in Aspirin-treated FAP patients with upregulated *VIM* (group #1) and downregulated *HBB* (Group #2), values are reported as scatter dot plots with mean + SD, *n* = 3 each group. (**C**–**E**) A two-tailed Student’s *t*-test analyzed the two sets of data. * *p* < 0.05, ** *p* < 0.01 vs. VIM (panel **C**) or group #1 (panel (**D**,**E**)).

Considering the proteins detected both at baseline and after the Aspirin treatment, only 8 proteins were changed in >50% of the six individuals studied (Figure 10A). No network was identified among these proteins (using STRING v11) (Figure 10A). However, only for HBB, VIM, and FAM3D, the fold change vs. baseline was statistically significant (Figure 10A). Based on residual urinary 11-dehydro-TXB_2_ levels measured after the Aspirin treatment, the six individuals were divided into two groups: Group #1 showed significantly higher values than Group #2 (Figure 10B). Interestingly, those of Group #1 showed upregulation of VIM vs. baseline, while those of Group #2 showed downregulation of HBB (Figure 10C and Figure S3). These changes were validated by qPCR analysis (Figure 10D). Among the clinical and demographic parameters that characterized the two groups of individuals, we found that the Aspartate aminotransferase (AST)/Alanine aminotransferase (ALT) ratio was significantly different (Figure 10B,E and Figure S4). Moreover, Group #1 showed a higher absolute number of lymphocytes and neutrophils in the peripheral blood than Group #2 (Figure S5). A significant linear relationship was found between the fold changes of adenoma FAM3D and urinary levels of 11-dehydro-TXB_2_ (r^2^ = 0.891; *p* = 0.0314) (Figure S6).

## 4. Discussion

The role of Aspirin as an anticancer agent in FAP is uncertain. In this setting, we performed a biomarker-based clinical study to explore on-target (i.e., COX pathways) and off-target effects of the chronic dosing with low-dose Aspirin.

In FAP patients, we found enhanced systemic biosynthesis of TXA_2_, PGE_2_, PGI_2_, and PGD_2_ vs. healthy individuals matched for age and sex. The coordinated increase in the systemic biosynthesis of all four main prostanoids suggests the contribution of enhanced arachidonic acid (AA) availability, possibly via cytosolic phospholipase A_2_ (cPLA_2_α), which hydrolyzes AA from cellular membrane phospholipids, thus providing the substrate to COX-isozymes [25]. Enhanced expression of *Pla2g4a* was detected in the adenomas of *Apc^Min/+^* mice (an animal model of FAP [23,24]) vs. normal mucosa of *WT* mice, accompanied by the overexpression of the inflammatory cytokine IL-6. Notably, IL-6 and STAT3 activation lead to the induction of cPLA_2_ expression [26].

Low-dose Aspirin caused an almost complete suppression of platelet TXA_2_ biosynthesis ex vivo, thus abrogating the contribution of platelets to the systemic TXA_2_ generation. In FAP patients chronically treated with low-dose Aspirin, residual urinary 11-dehydro-TXB_2_ not inhibited by the drug (i.e., the extraplatelet contribution of systemic TXA_2_ biosynthesis) was comparable to the values detected in healthy controls at baseline. However, in FAP patients treated with low-dose Aspirin, residual 11-dehydro-TXB_2_ (0.450 ± 0.250 ng/mg creatinine) was approximately 5-fold higher (*p* < 0.01) than previously detected in healthy subjects and individuals undergoing CRC screening treated with the drug at the same dose (0.091 ± 0.055 and 0.091 ± 0.037 ng/mg creatinine, respectively) (Figure S7) [13,14]. The systemic TXA_2_ biosynthesis unaffected by Aspirin can derive from the colorectal tissue of FAP patients, which express high levels of COX-1 (only partially acetylated by low-dose Aspirin) and TXS. Giardiello et al. [27] have reported that TXB_2_ is significantly elevated in the colorectal mucosa of FAP patients compared to healthy controls. Colorectal mucosa expressed TP receptors, thus suggesting that TXA_2_ biosynthesis, both in baseline conditions and after the Aspirin treatment, could play protumorigenic and proinflammatory effects. The knockdown of *TBXA2R* (TP receptor) or *TBXAS1* (TXS) in human colorectal cancer cells results in fewer colonies being formed in soft agar than in control cells [28]. We have recently shown that TXA_2_-dependent TP activation leads to the induction of COX-2, proliferation, and migration of intestinal myofibroblasts, thus playing a crucial role in adenoma development and its growth [18,29,30].

PGE_2_ plays multifaceted roles in cancer [31,32,33], and the evaluation of urinary PGEM is a promising biomarker for prognosticating cancer risk and disease progression [34]. In FAP, we have previously shown that enhanced urinary PGEM is reduced by celecoxib (a selective COX-2 inhibitor), suggesting that it reflects the biosynthesis of PGE_2_ in vivo, mainly from COX-2 [35]. However, in the present study, COX-2 mRNA and protein were undetectable in the colorectal adenomas and normal tissue of FAP patients. It is plausible that COX-2 was induced in stromal cells of adenomas [36], but it was undetectable in our homogenate preparations. In adenomas of *Apc^Min/+^* mice, we detected enhanced COX-2 expression vs. normal tissue of *WT* mice by qPCR. In FAP patients, the COX-1/cPGES pathway is highly expressed and can contribute to enhanced urinary PGEM when cPLA_2_ is induced and 15-PGDH is repressed. Low-dose Aspirin only partially acetylated COX-1 in colorectal biopsies, thus explaining its failure to prevent the increase of systemic PGE_2_ generation.

Systemic PGI_2_ biosynthesis was enhanced in FAP patients mainly via COX-2 [37] since celecoxib profoundly reduced urinary PGIM in this setting [35]. Vascular cell PGI_2_ can contribute to urinary PGIM; however, polyp epithelium can generate this prostanoid [36,38]. Tumors derived from cells expressing PGI_2_ synthase (PGIS) grew lower and exhibited less abundant vasculature [39]. Low-dose Aspirin did not affect the enhanced PGIM levels detected in FAP, thus allowing this antitumor signaling pathway to be active.

Systemic biosynthesis of PGD_2_ was also enhanced in FAP patients vs. healthy controls, and low-dose Aspirin partially prevented it. PGD_2_ has been detected in the colorectal mucosa of FAP patients [27], but its role in CRC is contradictory [31].

Several lines of evidence have shown that Aspirin can influence COX-independent molecular pathways [40,41]. Thus, we carried out the proteomics profile of colorectal adenomas of FAP patients untreated and chronically treated with low-dose Aspirin. We identified 1436 proteins, and 884 were common at baseline and after the Aspirin treatment. Among the top-ranked KEGG pathways that describe the protein networks, inflammatory-driven diseases, viral carcinogenesis, and bacterial invasion epithelial cells are of interest. Among the proteins detected in adenomas at baseline and after the Aspirin treatment, the changes in vimentin and HBB (hemoglobin subunit beta) were the most relevant responses to the drug treatment. The two proteins were uniquely upregulated or downregulated, respectively, in two different groups of patients. The downregulation of HBB by Aspirin could be beneficial. It has been reported that the depletion of HBB in circulating tumor cells (CTC) increases apoptosis and reduces CTC-derived lung metastases [42]. Our results provide the rationale to explore the role of HBB in colorectal adenoma development and the mechanism of Aspirin modulation. Differently, the upregulation of vimentin in the adenomas of Aspirin-treated FAP patients is an unfavorable effect. It has been shown that vimentin contributes to tumorigenesis, metastasis, invasion, and therapeutic resistance of various tumors [43]. The upregulation of vimentin in adenomas of some Aspirin-treated FAP patients could identify nonresponders to Aspirin. Notably, higher residual urinary 11-dehydro-TXB_2_ levels characterized these individuals. Since appropriate inhibition of platelet COX-1 activity by Aspirin was found (serum TXB_2_ was comparably reduced by more than 98%), colorectal tissue plausibly contributed to enhanced residual systemic TXA_2_ biosynthesis in these individuals. Enhanced colorectal TXA_2_ generation could contribute to the upregulation of vimentin in adenomas. We have previously shown that the TXA_2_ mimetic U46619 induces vimentin in intestinal myofibroblasts [30].

In the same individuals treated with Aspirin, FAM3D resulted upregulated (Figure S3). A significant linear relationship was found between the fold changes of adenoma FAM3D and residual urinary levels of 11-dehydro-TXB_2_ (r^2^ = 0.891; *p* = 0.0314) (Figure S6). Protein FAM3D belongs to a cytokine-like family and is involved in gastrointestinal-related inflammation processes. FAM3D is upregulated in the colon of the dextran sulfate sodium-induced colitis mouse model [44]. FAM3D has a high affinity with formyl peptide receptors 1 and 2, which are highly expressed in leukocytes [44]. Whether TXA_2_ regulates FAM3D expression in colorectal adenomas is unknown and deserves further investigation. Notably, *FAM3D* is a PPARα target gene [45], and it is known that some eicosanoids and fatty acids, including AA, can bind directly to PPARα [46].

Interestingly the FAP group showing higher residual urinary 11-dehydro-TXB_2_ values was also characterized by a higher AST/ALT ratio (De Ritis ratio). The serum AST/ALT ratio is a measure of liver injury but is associated with some chronic diseases and mortality [47]. It has been found to represent a prognostic marker for cancer development and disease-free survival in nonmetastatic colorectal cancer patients at stages II and III [48]. Our findings may implicate enhanced systemic inflammation in FAP patients with a higher AST/ALT ratio. Xu et al. have reported that AST/ALT ratio was positively correlated with circulating inflammatory cytokines, such as TNF-α, IL-4, and IL-6, in type 2 diabetes mellitus patients [49]. In the present study, FAP patients with a higher AST/ALT ratio showed a higher count of blood leukocytes (mainly neutrophils and lymphocytes). In these FAP patients, enhanced TXA_2_ biosynthesis in colorectal adenomas could contribute to an inflammatory microenvironment playing a pivotal role in tumorigenesis. In this scenario, low-dose Aspirin was ineffective due to its limited capacity to acetylate colorectal adenoma COX-1.

Low-dose Aspirin can exert antitumor effects by inhibiting platelet-dependent TXA_2_ production. This hypothesis is sustained by the finding that *Apc^Min/+^* mice with the specific deletion of platelet COX-1 have a reduced number (67%) and size (32%) of tumors in the small intestine [18].

The limitation of our study is the small sample size, but FAP is a rare inherited condition, and we aimed to select patients without risk factors for cardiovascular disease and smoking habits, which reduced the capacity to enroll patients in the study. The strength of this study relates to performing deep phenotyping of FAP patients treated with low-dose Aspirin using innovative biomarkers of drug action and proteome analysis of colorectal adenomas. However, larger sample-size studies in FAP patients treated with Aspirin should incorporate the assessment of the proteomics of colorectal adenomas to confirm the changes found in the present study.

## 5. Conclusions

Our data identify the sources of variability in the antitumor effects of Aspirin in FAP. Although low-dose Aspirin appropriately inhibited the platelet, persistently high levels of systemic TXA_2_ and PGE_2_ biosynthesis were found, plausibly for a marginal inhibitory effect of the drug on prostanoid biosynthesis in the colorectum. Our findings provide the rationale for novel chemotherapeutic strategies in FAP by blocking the effects of both platelet and colorectal TXA_2_ with TP antagonists [50]. Ifetroban (a TP antagonist) is currently being evaluated in a human clinical trial to suppress cancer metastases (ClinicalTrials.gov Identifier: NCT03694249). Furthermore, blocking PGE_2_ signaling with EP2/EP4 receptor antagonists might represent an appealing anti-inflammatory and antitumor strategy that is under clinical evaluation (ClinicalTrials.gov Identifier: NCT04344795).

## Figures and Tables

**Table 1:** Baseline characteristics of FAP patients and healthy subjects.

	FAP Patients (*n* = 8)	Healthy Subjects (*n* = 8)
Age, y	40.25 ± 8.73	41.00 ± 10.36
Sex, % female	75.00	75.00
BMI, kg/m^2^	24.17 ± 3.45	23.03 ± 2.85
Systolic blood pressure, mmHg	110.80 ± 8.19	114.40 ± 8.21
Diastolic blood pressure, mmHg	69.00 ± 5.24	72 ± 3.85
Platelets count (×10^3^/mL)	265 ± 97.17	252.40 ± 43.18
Hematocrit (%)	42.71 ± 1.88	41.89 ± 2.21
Haemoglobin (g/dL)	14.26 ± 0.89	13.77 ± 0.79
Glycemia, mg/dL	86.13 ± 10.01	92.00 ± 8.67
Creatinine, mg/dL	0.69 ± 0.16	0.80 ± 0.10
Total cholesterol, mg/dL	195.40 ± 30.80	196.00 ± 27.36
Type of polyps		
Serrated adenoma with low-grade dysplasia, n (%)	1 (12.5)	0 (0)
Tubular adenoma with low-grade dysplasia, n (%)	3 (37.5)	0 (0)
Tubulovillous adenoma with low-grade dysplasia, n (%)	4 (50)	0 (0)
Mutations		
APC, n (%)	7 (87.5)	0 (0)
MUTYH, n (%)	1 (12.5)	0 (0)
Classification		
Intermediate, n (%)	5 (62.5)	0 (0)
Attenuated, n (%)	3 (37.5)	0 (0)

Data are expressed as mean ± SD; abbreviations: MutY human homolog (MYH), adenomatous polyposis coli (APC), not determined (ND).

## Data Availability

The raw data supporting the conclusion of this article will be made available by the authors without undue reservation. Mass spectrometry data have been submitted through the MassIVE (mass spectrometry interactive virtual environment) repository and are available with the following identifier: MSV000091536 (ftp://MSV000091536@massive.ucsd.edu). The dataset will be made publicly available in ProteomeXchange after manuscript publication with the identifier PXD041059.

## Supplementary Materials

The following supporting information can be downloaded at: https://www.mdpi.com/article/10.3390/cancers15092457/s1, Supplementary methods; Figure S1. Uncropped gels of Western blotting membrane presented in Figure 4A; Figure S2. Uncropped gels of Western blotting shown in Figure 4B; Figure S3. Heat map of fold-change of adenoma proteins in six Aspirin-treated FAP patients vs. predrug (baseline); Figure S4. Clinical and demographic features of FAP patients with upregulated VIM (group #1) and downregulated HBB (group #2); Figure S5. Blood cells counted in FAP patients with upregulated VIM (group #1) and downregulated HBB (group #2). Figure S6. Simple linear regression between residual 11-dehydro-TXB_2_ and fold-change of colorectal adenoma FAM3D in aspirin-treated FAP patients vs. predrug; Figure S7. Urinary 11-dehydro-TXB2 in healthy subjects and individuals undergoing CRC screening treated with low-dose Aspirin for a week, previously published; Table S1. List of all identified proteins in tissue biopsies from FAP patients.
