# Supplementary material for: Biomarkers of Response to Low-Dose Aspirin in Familial Adenomatous Polyposis Patients
Source: Cancers (Basel). 2023 Apr 25;15(9):2457. doi: 10.3390/cancers15092457 (PMC10177499; doi:10.3390/cancers15092457)
Supplement: Supplementary file 1 [file cancers-15-02457-s001.zip › cancers-2338240-supplementary.pdf]

Supplementary Material

Biomarkers of response to low-dose Aspirin in Familial Adenomatous Polyposis patients

Angel Lanas, Stefania Tacconelli, Annalisa Contursi, Elena Piazuelo, Annalisa Bruno, Maurizio Ronci, Simone Marcone, Melania Dovizio, Federico Sopeña, Lorenza Falcone, Cristina Milillo, Matteo Mucci, Patrizia Ballerini, Paola Patrignani

Supplementary Methods

Clinical study

Study design

Day 0 (baseline visit). Following confirmation of each patient's suitability for the study (based on inclusion criteria and absence of exclusion criteria), patients were invited to participate in the study; after signing informed consent, the following procedures were performed:

- urinary pregnancy test (women of childbearing age were required to have a negative pregnancy test within 24-hour before the first dose of the study drug);
- blood extraction for biochemical and hematological analysis
- medical history and physical examination, including vital signs (blood pressure, heart rate, and lung efficiency);
- delivery of containers for 24-hour urine collections.

Day 1. At 8:00 a.m., FAP patients went to the hospital. The patients delivered urine collected in the previous 24 hour. A venous blood sample (30 ml) was taken to conduct some laboratory tests and obtain serum samples for TXB_2 assessment and washed platelets to evaluate COX-1 acetylation. Immediately after, a colonoscopy was performed, and polyps, and normal tissue of the rectum, left and right colon were collected; they were washed in phosphate buffer solution, immediately placed into a tube, frozen in liquid nitrogen, and stored at -80°C until

analysis. Then, the patient took an Aspirin tablet [100 mg, enteric-coated(EC) formulation, Adiro, Bayer] under fasting conditions. A box with 89 Aspirin tablets was provided to each patient. Serum samples, washed platelets, and urine samples were stored at-80°C before the assessment of biomarkers.

Day 2 to Day 89. At 8:00 a.m., each patient took the Aspirin tablet at home under fasting conditions.

Day 90. At 8:00 a.m., FAP patients took the last Aspirin tablet at home under fasting conditions and started the 24-hour urine collection.

Day 91 (Visit 2). FAP patients returned to the clinic with a 24-hour urine collection, and a venous blood sample (30 ml) was taken to obtain serum samples and washed platelets; moreover, colorectal biopsies of normal mucosa and colorectal polyps were collected, as described on day 0. Patients returned the box for compliance assessment by tablet counts. Tablet count adherence was 100%, and no enrolled subject was excluded from the study. In addition, compliance was monitored by contacting the subjects by telephone every 2 weeks. The polyps and tissue samples were processed and analyzed by the Laboratories of Pathology of CIBA and the University Clinic Hospital Lozano Blesa (Zaragoza, Spain).

Inclusion and exclusion criteria

The individuals were included in the study if these criteria were satisfied: i) age 18-65 years; ii) established diagnosis of FAP according to current diagnostic criteria by genotype and phenotype. The exclusion criteria were: i) allergy to Aspirin or other nonsteroidal anti-inflammatory drugs (NSAIDs); ii) previous use of Aspirin, or other NSAIDs, or misoprostol in the previous 30 days and/or anticipated need for these drugs during the study period; iii) pregnant women; iv) breastfeeding; v) other malignancies (excluding CRC or FAP related tumors) diagnosed in the previous five years; vi) severe comorbid conditions, including pulmonary, cardiac (and cardiovascular risk factors), liver or kidney diseases; vii) peptic ulcer

history or any other gastrointestinal disease that could be considered a contraindication for aspirin use without the concomitant use of a proton-pump inhibitor. All individuals should not be smokers.

Patients did not take any medications before enrolment. During the study, only paracetamol was allowed as analgesic drug for not more than 10 days: only 1 patient have assumed paracetamol 1000 mg for 9 days.

Western blot analysis

Washed platelets, colorectal polyps, and normal tissue samples from the rectum, left, and right colon were obtained as previously described [13, 14]; they were washed in PBS and immediately placed into a tube and frozen in liquid nitrogen. The specimens were stored at -80°C until the analysis. The samples were homogenized as previously reported [13, 14~~, 51~~]. The protein concentration of homogenates was assessed by the Bradford method (Bio-Rad, Milan, Italy) and aliquots were loaded onto Sodium Dodecyl SulphatePolyAcrylamide Gel Electrophoresis (SDS-PAGE) (9-15%, depending on the MW of the protein). Separated proteins were transferred to the PVDF membrane (Bio-Rad). The membrane was saturated with 5% non-fat milk solution in Tris-buffered saline-0.1% Tween-20 (TBS-Tween20). COX-2 protein expression of colorectal biopsies and adenomas was assessed by cutting the membranes into two parts at the visible 50 kDa protein molecular weight (MW) marker band. The membranes above the 50 kDa band were incubated with COX-2 monoclonal antibody [Cayman Chemical (item#160112), Denver, Colorado; diluted 1:1000 in TBS-Tween20], while the lower half of the membranes were probed with GAPDH monoclonal antibody [Sigma Aldrich (item#G8795) diluted 1:4000 in TBS-Tween20] overnight at 4°C. Another blot was performed to assess COX-1 protein expression in the same biological samples following the same procedure described above but using COX-1 polyclonal antibodies [Cayman Chemical (item#160108); diluted 1:1000 in TBS-Tween20]; then, the membrane

was washed three times (for 30 minutes) with TBS-Tween20 and then incubated with the GAPDH monoclonal antibody overnight at 4°C. For the assessment of TXS (TXA_2 synthase) protein, the membrane was incubated with primary antibodies [TXA_2 synthase polyclonal antibodies (Cayman Chemical, item#160715; diluted 1:200 in TBS-Tween20, 5% milk)] overnight at 4°C; then, the membrane was washed three times (for 30 minutes) with TBS-Tween20 and then incubated with the GAPDH monoclonal antibody overnight at 4°C. Another blot was performed to assess TP (TXA_2 receptor) protein; the membrane was incubated with TP polyclonal antibody (Cayman Chemical, item#10004452, diluted 1:200 in TBS-Tween 20, 5% milk)] overnight at 4°C; then, the membrane was washed three times (for 30 minutes) with TBS-Tween20 and then incubated with the GAPDH monoclonal antibody overnight at 4°C.

For the analysis of expression of TXS and TP in platelets, PVDF membranes were incubated with the TP receptor polyclonal antibodies (reported above) overnight at 4°C; then, the membranes were washed three times (for 30 minutes) with TBS-Tween20 and incubated with COX-1 polyclonal antibodies (described above) overnight at 4°C. Finally, to detect the loading control GAPDH, the membranes were washed three times (for 30 minutes) with TBS-Tween20 and then incubated with the GAPDH monoclonal antibody overnight at 4°C.

To detect the specific protein bands, after the incubation with primary antibodies, membranes were washed three times (10 minutes) in TBS-Tween20 and incubated with an appropriate dilution of HRP-conjugated secondary antibodies (for COX-1, TXS, and TP from Sigma, item#A0545; for COX-2 and GAPDH from Calbiochem, item#401253) for 1 hour at room temperature. Following washing, immunoreactive bands were detected by incubation of membranes with ECL reagent (GE Healthcare, Illinois, USA) and scanning using Alliance 1D software (UVITEC, Cambridge, UK) and normalized to the OD (optical density) of GAPDH.

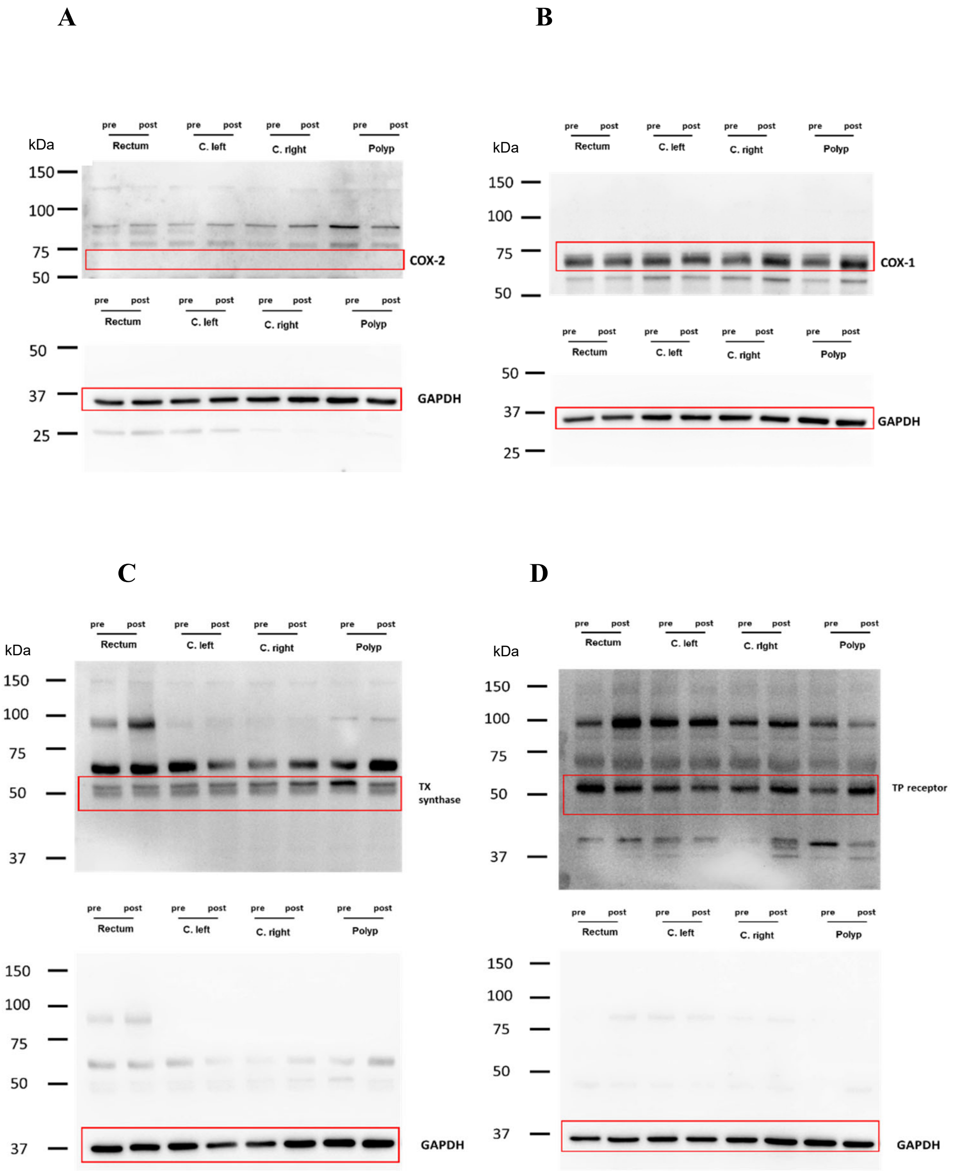

Figure S1. Uncropped gels of Western blotting membrane presented in Figure. 4A. **(A, B)** The membranes were cut into two parts at the visible 50 kDa protein MW marker band as described in Supplementary Material. The membranes above 50 kDa band were probed with COX-2 or COX-1 antibodies, while the lower half of the membranes were probed with GAPDH antibodies. **(C, D)** The membranes were probed respectively with a TX synthase antibody or a TP receptor antibody and after an extensive washing the membranes were probed with GAPDH polyclonal antibodies. Some nonspecific bands at higher MW were detected. The red boxes underline the areas presented in Figure 4A.

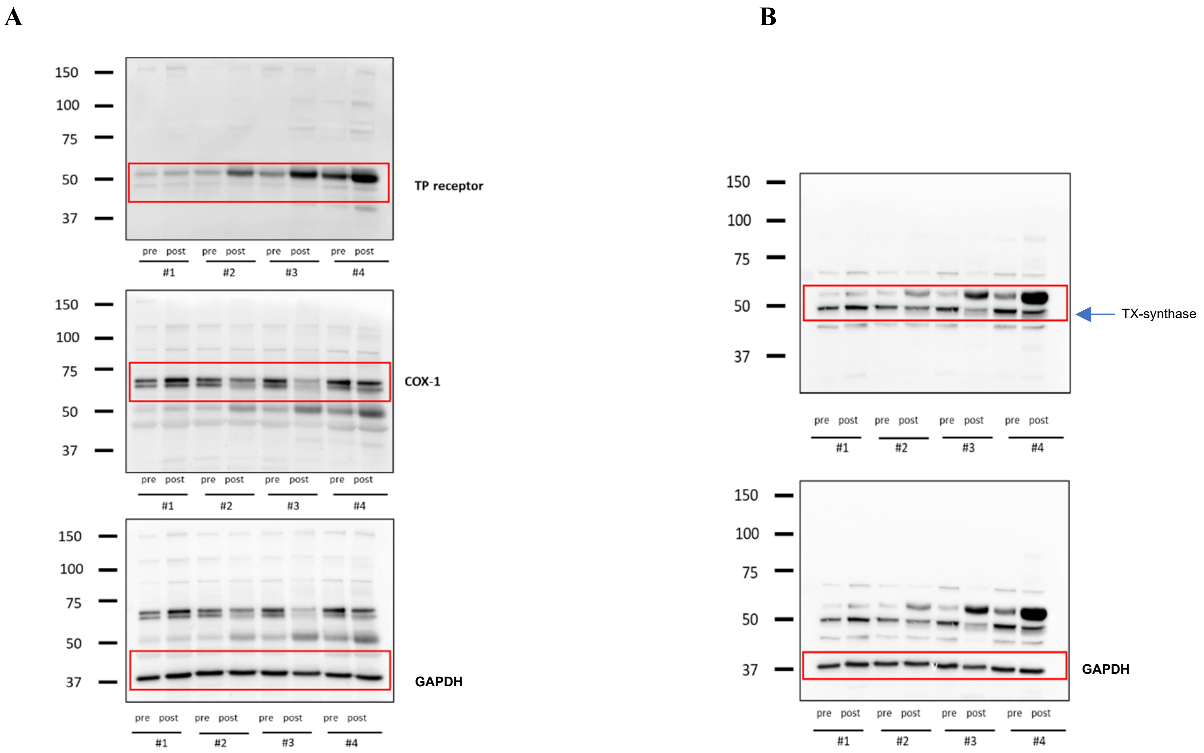

Figure S2. Uncropped gels of Western blotting presented in Figure 4B. **(A)** The membranes were probed with a TP receptor monoclonal antibody; after extensive washing, they were incubated with a COX-1 monoclonal antibody and then with a GAPDH polyclonal antibody as reported in Supplementary Material. **(B)** The membranes were incubated with a TX synthase monoclonal antibody and, after extensive washing, were probed with GAPDH polyclonal antibody as reported in Supplementary Material. The red boxes underline the areas presented in Figure 4B. Some nonspecific bands at higher MW were detected. The arrow indicated the protein with appropriate MW (i.e., 50 kDa) of TXS used for OD analysis.

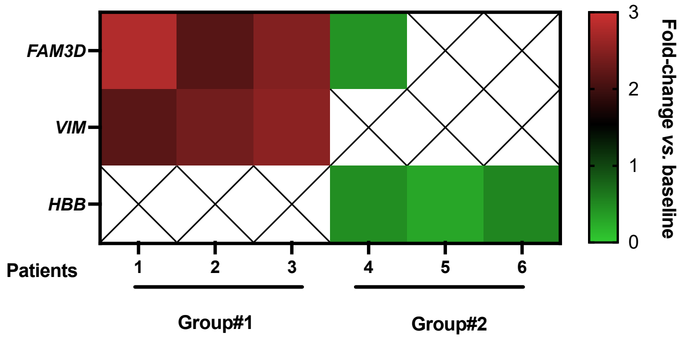

Figure S3. Heat map of fold-change of adenoma proteins in six Aspirin-treated FAP patients *vs*. predrug (baseline). Group#1 and group#2 describe the patients with higher and lower residual urinary 11-dehydro-TXB_2 detected in FAP patients treated with low-dose Aspirin.

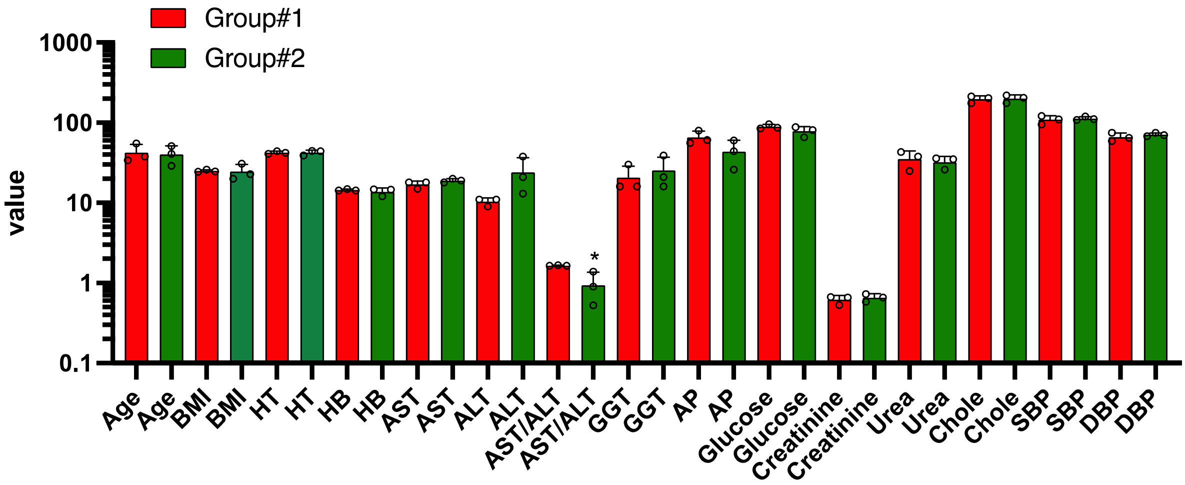

Figure S4. Clinical and demographic features of FAP patients with upregulated VIM (group #1) and downregulated HBB (group #2). Values are reported as scatter dot plots with mean+SD, n=3 each group.*P<0.05 *vs*. the group#1. A two-tailed Student's test analyzed the 2 sets of data. Abbreviations and unit of measurement: Age (years), Body Mass Index (BMI, kg/m^2), Hematocrit (HT, %), Hemoglobin (HB, g/dl), Aspartate aminotransferase (AST, U/l), Alanine aminotransferase (ALT, U/l), Gamma-glutamyltransferase (GGT, U/l), Alkaline Phosphatase (AP, U/l), Glucose (mg/dl), Creatinine (mg/dl), Urea (mg/dl), Cholesterol (mg/dl), Systolic blood pressure (SBP, mmHg), Diastolic blood pressure (DBP, mmHg).

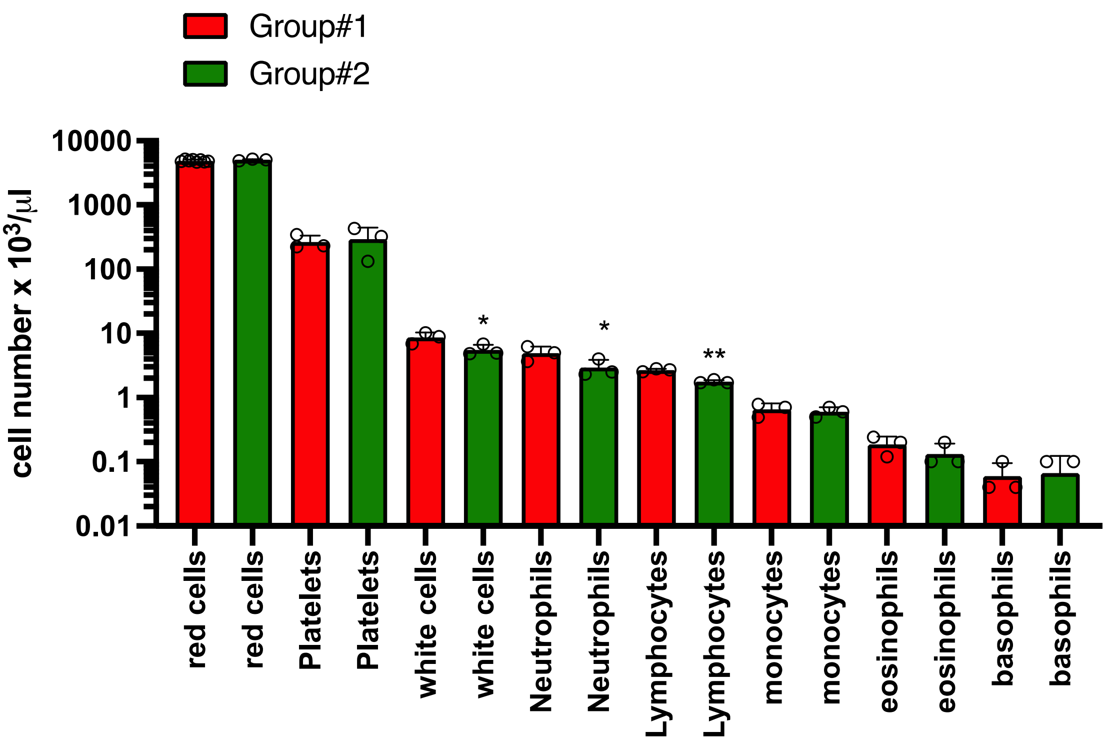

Figure S5. Blood cells counted in FAP patients with upregulated VIM (group #1) and downregulated HBB (group #2). Values are reported as scatter dot plots with mean+SD, n=3 each group. *P<0.05, **P<0.01 *vs*. the group#1. A two-tailed Student's test analyzed the 2 sets of data.

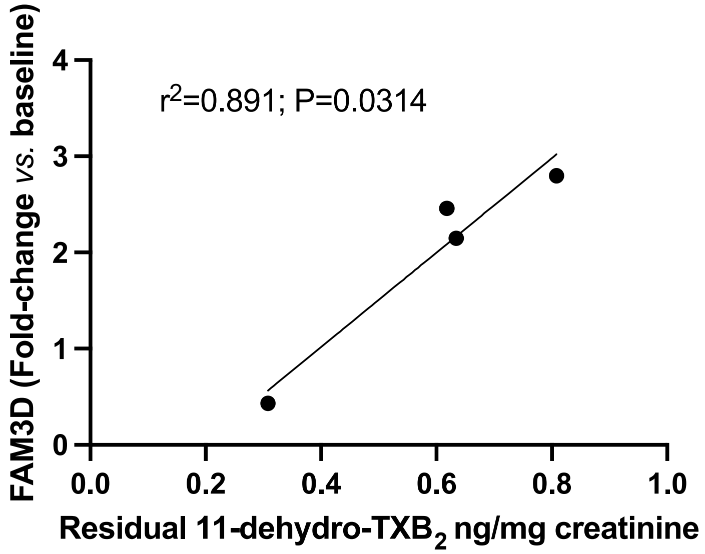

Figure S6. Simple linear regression between residual 11-dehydro-TXB2 and fold-change of colorectal adenoma FAM3D in aspirin-treated FAP patients *vs*. predrug.

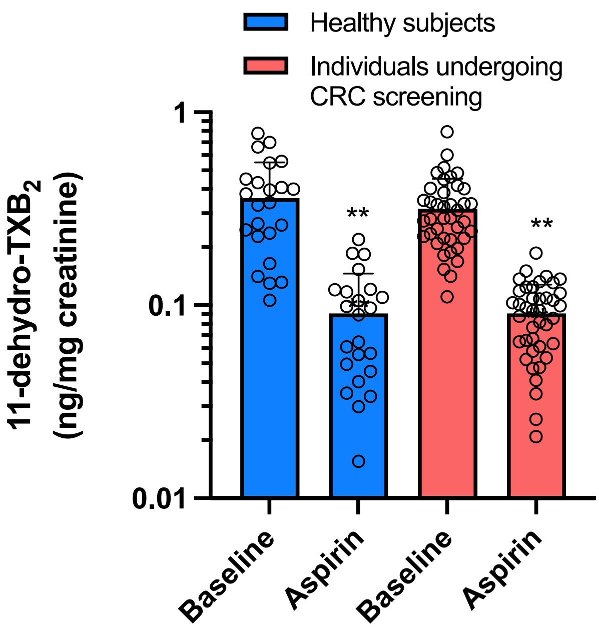

Figure S7. Urinary 11-dehydro-TXB_2 in healthy subjects and individuals undergoing CRC screening treated with low-dose Aspirin for a week, previously published [13, 14]**.** All values are shown as scatter dot plots with mean+SD and analyzed by one-way ANOVA followed by Tukey's multiple comparisons test. **P<0.01 *vs.* its own baseline.

Table S1. List of all identified proteins in tissue biopsies from FAP patients

**Protein ID**	**David Gene Name**
A1L0T0	ilvB acetolactate synthase like(ILVBL)
A3KMH1	von Willebrand factor A domain containing 8(VWA8)
A5A3E0	POTE ankyrin domain family member F(POTEF)
A6H8Y1	B double prime 1, subunit of RNA polymerase III transcription initiation factor IIIB(BDP1)
A6NCM1	IQ motif containing with AAA domain 1 like(IQCA1L)
A6NHG4	D-dopachrome tautomerase like(DDTL)
A6NIZ1	RAP1B like (pseudogene)(RAP1BL)
A6NNZ2	tubulin beta 8B(TUBB8B)
A8K7I4	chloride channel accessory 1(CLCA1)
A8MWD9	small nuclear ribonucleoprotein polypeptide G pseudogene 15(SNRPGP15)
B2RPK0	high mobility group box 1 pseudogene 1(HMGB1P1)
B5MCN3	SEC14 like lipid binding 6(SEC14L6)
B5ME19	eukaryotic translation initiation factor 3 subunit C like(EIF3CL)
B9A064	immunoglobulin lambda like polypeptide 5(IGLL5)
E7EW31	proline rich basic protein 1(PROB1)
E9PAV3	nascent polypeptide associated complex subunit alpha(NACA)
I6L899	golgin A8 family member R(GOLGA8R)
O00115	deoxyribonuclease 2, lysosomal(DNASE2)
O00148	DExD-box helicase 39A(DDX39A)
O00151	PDZ and LIM domain 1(PDLIM1)
O00159	myosin IC(MYO1C)
O00182	galectin 9(LGALS9)
O00186	syntaxin binding protein 3(STXBP3)
O00203	adaptor related protein complex 3 subunit beta 1(AP3B1)

O00214	galectin 8(LGALS8)
O00231	proteasome 26S subunit, non-ATPase 11(PSMD11)
O00232	proteasome 26S subunit, non-ATPase 12(PSMD12)
O00264	progesterone receptor membrane component 1(PGRMC1)
O00299	chloride intracellular channel 1(CLIC1)
O00303	eukaryotic translation initiation factor 3 subunit F(EIF3F)
O00391	quiescin sulfhydryl oxidase 1(QSOX1)
O00423	EMAP like 1(EML1)
O00461	golgi integral membrane protein 4(GOLIM4)
O00487	proteasome 26S subunit, non-ATPase 14(PSMD14)
O00534	von Willebrand factor A domain containing 5A(VWA5A)
O00571	DEAD-box helicase 3 X-linked(DDX3X)
O00584	ribonuclease T2(RNASET2)
O00748	carboxylesterase 2(CES2)
O00754	mannosidase alpha class 2B member 1(MAN2B1)
O00764	pyridoxal kinase(PDXK)
O14556	glyceraldehyde-3-phosphate dehydrogenase, spermatogenic(GAPDHS)
O14579	COPI coat complex subunit epsilon(COPE)
O14617	adaptor related protein complex 3 subunit delta 1(AP3D1)
O14647	chromodomain helicase DNA binding protein 2(CHD2)
O14686	lysine methyltransferase 2D(KMT2D)
O14773	tripeptidyl peptidase 1(TPP1)
O14818	proteasome 20S subunit alpha 7(PSMA7)
O14867	BTB domain and CNC homolog 1(BACH1)
O14974	protein phosphatase 1 regulatory subunit 12A(PPP1R12A)
O14979	heterogeneous nuclear ribonucleoprotein D like(HNRNPDL)
O14983	ATPase sarcoplasmic/endoplasmic reticulum Ca2+ transporting 1(ATP2A1)
O15056	synaptojanin 2(SYNJ2)

O15090	zinc finger protein 536(ZNF536)
O15127	secretory carrier membrane protein 2(SCAMP2)
O15143	actin related protein 2/3 complex subunit 1B(ARPC1B)
O15144	actin related protein 2/3 complex subunit 2(ARPC2)
O15145	actin related protein 2/3 complex subunit 3(ARPC3)
O15213	WD repeat domain 46(WDR46)
O15260	surfeit 4(SURF4)
O15305	phosphomannomutase 2(PMM2)
O15371	eukaryotic translation initiation factor 3 subunit D(EIF3D)
O15372	eukaryotic translation initiation factor 3 subunit H(EIF3H)
O15382	branched chain amino acid transaminase 2(BCAT2)
O15400	syntaxin 7(STX7)
O15511	actin related protein 2/3 complex subunit 5(ARPC5)
O15523	DEAD-box helicase 3 Y-linked(DDX3Y)
O15533	TAP binding protein(TAPBP)
O43143	DEAH-box helicase 15(DHX15)
O43252	3'-phosphoadenosine 5'-phosphosulfate synthase 1(PAPSS1)
O43324	eukaryotic translation elongation factor 1 epsilon 1(EEF1E1)
O43390	heterogeneous nuclear ribonucleoprotein R(HNRNPR)
O43399	TPD52 like 2(TPD52L2)
O43432	eukaryotic translation initiation factor 4 gamma 3(EIF4G3)
O43488	aldo-keto reductase family 7 member A2(AKR7A2)
O43491	erythrocyte membrane protein band 4.1 like 2(EPB41L2)
O43684	BUB3 mitotic checkpoint protein(BUB3)
O43704	sulfotransferase family 1B member 1(SULT1B1)
O43707	actinin alpha 4(ACTN4)
O43765	small glutamine rich tetratricopeptide repeat co-chaperone alpha(SGTA)
O43776	asparaginyl-tRNA synthetase 1(NARS1)

O43852	calumenin(CALU)
O43865	adenosylhomocysteinase like 1(AHCYL1)
O60218	aldo-keto reductase family 1 member B10(AKR1B10)
O60234	glia maturation factor gamma(GMFG)
O60256	phosphoribosyl pyrophosphate synthetase associated protein 2(PRPSAP2)
O60282	kinesin family member 5C(KIF5C)
O60313	OPA1 mitochondrial dynamin like GTPase(OPA1)
O60361	NME2 pseudogene 1(NME2P1)
O60488	acyl-CoA synthetase long chain family member 4(ACSL4)
O60506	synaptotagmin binding cytoplasmic RNA interacting protein(SYNCRIP)
O60547	GDP-mannose 4,6-dehydratase(GMDS)
O60656	UDP glucuronosyltransferase family 1 member A9(UGT1A9)
O60664	perilipin 3(PLIN3)
O60701	UDP-glucose 6-dehydrogenase(UGDH)
O60716	catenin delta 1(CTNND1)
O60749	sorting nexin 2(SNX2)
O60763	USO1 vesicle transport factor(USO1)
O60814	H2B clustered histone 12(H2BC12)
O60844	zymogen granule protein 16(ZG16)
O60884	DnaJ heat shock protein family (Hsp40) member A2(DNAJA2)
O60888	cutA divalent cation tolerance homolog(CUTA)
O75083	WD repeat domain 1(WDR1)
O75106	amine oxidase copper containing 2(AOC2)
O75131	copine 3(CPNE3)
O75208	coenzyme Q9(COQ9)
O75223	gamma-glutamylcyclotransferase(GGCT)
O75323	nipsnap homolog 2(NIPSNAP2)
O75340	programmed cell death 6(PDCD6)

O75347	tubulin folding cofactor A(TBCA)
O75348	ATPase H+ transporting V1 subunit G1(ATP6V1G1)
O75356	ectonucleoside triphosphate diphosphohydrolase 5 (inactive)(ENTPD5)
O75363	brain enriched myelin associated protein 1(BCAS1)
O75367	macroH2A.1 histone(MACROH2A1)
O75368	SH3 domain binding glutamate rich protein like(SH3BGRL)
O75369	filamin B(FLNB)
O75380	NADH:ubiquinone oxidoreductase subunit S6(NDUFS6)
O75390	citrate synthase(CS)
O75396	SEC22 homolog B, vesicle trafficking protein(SEC22B)
O75431	metaxin 2(MTX2)
O75436	VPS26 retromer complex component A(VPS26A)
O75439	peptidase, mitochondrial processing subunit beta(PMPCB)
O75477	ER lipid raft associated 1(ERLIN1)
O75489	NADH:ubiquinone oxidoreductase core subunit S3(NDUFS3)
O75643	small nuclear ribonucleoprotein U5 subunit 200(SNRNP200)
O75691	UTP20 small subunit processome component(UTP20)
O75795	UDP glucuronosyltransferase family 2 member B17(UGT2B17)
O75828	carbonyl reductase 3(CBR3)
O75874	isocitrate dehydrogenase (NADP(+)) 1(IDH1)
O75891	aldehyde dehydrogenase 1 family member L1(ALDH1L1)
O75915	ADP ribosylation factor like GTPase 6 interacting protein 5(ARL6IP5)
O75947	ATP synthase peripheral stalk subunit d(ATP5PD)
O75964	ATP synthase membrane subunit g(ATP5MG)
O94760	dimethylarginine dimethylaminohydrolase 1(DDAH1)
O94804	serine/threonine kinase 10(STK10)
O94826	translocase of outer mitochondrial membrane 70(TOMM70)
O94832	myosin ID(MYO1D)

O94905	ER lipid raft associated 2(ERLIN2)
O94979	SEC31 homolog A, COPII coat complex component(SEC31A)
O95154	aldo-keto reductase family 7 member A3(AKR7A3)
O95299	NADH:ubiquinone oxidoreductase subunit A10(NDUFA10)
O95336	6-phosphogluconolactonase(PGLS)
O95394	phosphoglucomutase 3(PGM3)
O95571	ETHE1 persulfide dioxygenase(ETHE1)
O95573	acyl-CoA synthetase long chain family member 3(ACSL3)
O95678	keratin 75(KRT75)
O95810	caveolae associated protein 2(CAVIN2)
O95831	apoptosis inducing factor mitochondria associated 1(AIFM1)
O95834	EMAP like 2(EML2)
O95861	3'(2'), 5'-bisphosphate nucleotidase 1(BPNT1)
O95865	dimethylarginine dimethylaminohydrolase 2(DDAH2)
O95994	anterior gradient 2, protein disulphide isomerase family member(AGR2)
O95996	APC regulator of WNT signaling pathway 2(APC2)
O96000	NADH:ubiquinone oxidoreductase subunit B10(NDUFB10)
P00325	alcohol dehydrogenase 1B (class I), beta polypeptide(ADH1B)
P00326	alcohol dehydrogenase 1C (class I), gamma polypeptide(ADH1C)
P00338	lactate dehydrogenase A(LDHA)
P00352	aldehyde dehydrogenase 1 family member A1(ALDH1A1)
P00367	glutamate dehydrogenase 1(GLUD1)
P00387	cytochrome b5 reductase 3(CYB5R3)
P00390	glutathione-disulfide reductase(GSR)
P00403	mitochondrially encoded cytochrome c oxidase II(MT-CO2)
P00403	MT-CO2 pseudogene 12(MTCO2P12)
P00441	superoxide dismutase 1(SOD1)
P00488	coagulation factor XIII A chain(F13A1)

P00491	purine nucleoside phosphorylase(PNP)
P00505	glutamic-oxaloacetic transaminase 2(GOT2)
P00558	phosphoglycerate kinase 1(PGK1)
P00738	haptoglobin(HP)
P00915	carbonic anhydrase 1(CA1)
P00918	carbonic anhydrase 2(CA2)
P00966	argininosuccinate synthase 1(ASS1)
P01009	serpin family A member 1(SERPINA1)
P01023	alpha-2-macroglobulin(A2M)
P01024	complement C3(C3)
P01034	cystatin C(CST3)
P01042	kininogen 1(KNG1)
P01591	joining chain of multimeric IgA and IgM(JCHAIN)
P01594	immunoglobulin kappa variable 1-33(IGKV1-33)
P01602	immunoglobulin kappa variable 1-5(IGKV1-5)
P01624	immunoglobulin kappa variable 3-15(IGKV3-15)
P01714	immunoglobulin lambda variable 3-19(IGLV3-19)
P01742	immunoglobulin heavy variable 1-69(IGHV1-69)
P01743	immunoglobulin heavy variable 1-46(IGHV1-46)
P01762	immunoglobulin heavy variable 3-11(IGHV3-11)
P01764	immunoglobulin heavy variable 3-23(IGHV3-23)
P01766	immunoglobulin heavy variable 3-13(IGHV3-13)
P01833	polymeric immunoglobulin receptor(PIGR)
P01834	immunoglobulin kappa constant(IGKC)
P01857	immunoglobulin heavy constant gamma 1 (G1m marker)(IGHG1)
P01859	immunoglobulin heavy constant gamma 2 (G2m marker)(IGHG2)
P01860	immunoglobulin heavy constant gamma 3 (G3m marker)(IGHG3)
P01861	immunoglobulin heavy constant gamma 4 (G4m marker)(IGHG4)

P01871	immunoglobulin heavy constant mu(IGHM)
P01876	immunoglobulin heavy constant alpha 1(IGHA1)
P01877	immunoglobulin heavy constant alpha 2 (A2m marker)(IGHA2)
P01889	major histocompatibility complex, class I, B(HLA-B)
P01893	major histocompatibility complex, class I, H (pseudogene)(HLA-H)
P01903	major histocompatibility complex, class II, DR alpha(HLA-DRA)
P02042	hemoglobin subunit delta(HBD)
P02070	hemoglobin, beta(HBB)
P02533	keratin 14(KRT14)
P02535	keratin 10(Krt10)
P02538	keratin 6A(KRT6A)
P02545	lamin A/C(LMNA)
P02584	profilin 1(PFN1)
P02647	apolipoprotein A1(APOA1)
P02671	fibrinogen alpha chain(FGA)
P02675	fibrinogen beta chain(FGB)
P02676	fibrinogen beta chain(FGB)
P02679	fibrinogen gamma chain(FGG)
P02749	apolipoprotein H(APOH)
P02763	orosomucoid 1(ORM1)
P02765	alpha 2-HS glycoprotein(AHSG)
P02768	albumin(ALB)
P02769	albumin(ALB)
P02774	GC vitamin D binding protein(GC)
P02786	transferrin receptor(TFRC)
P02787	transferrin(TF)
P02790	hemopexin(HPX)
P02795	metallothionein 2A(MT2A)

P04040	catalase(CAT)
P04066	alpha-L-fucosidase 1(FUCA1)
P04075	aldolase, fructose-bisphosphate A(ALDOA)
P04080	cystatin B(CSTB)
P04083	annexin A1(ANXA1)
P04179	superoxide dismutase 2(SOD2)
P04259	keratin 6B(KRT6B)
P04264	keratin 1(KRT1)
P04406	glyceraldehyde-3-phosphate dehydrogenase(GAPDH)
P04424	argininosuccinate lyase(ASL)
P04632	calpain small subunit 1(CAPNS1)
P04732	metallothionein 1E(MT1E)
P04733	metallothionein 1F(MT1F)
P04746	amylase alpha 2A(AMY2A)
P04792	heat shock protein family B (small) member 1(HSPB1)
P04843	ribophorin I(RPN1)
P04844	ribophorin II(RPN2)
P04899	G protein subunit alpha i2(GNAI2)
P04908	H2A clustered histone 8(H2AC8)
P04908	H2A clustered histone 4(H2AC4)
P05023	ATPase Na+/K+ transporting subunit alpha 1(ATP1A1)
P05026	ATPase Na+/K+ transporting subunit beta 1(ATP1B1)
P05091	aldehyde dehydrogenase 2 family member(ALDH2)
P05109	S100 calcium binding protein A8(S100A8)
P05141	solute carrier family 25 member 5(SLC25A5)
P05165	propionyl-CoA carboxylase subunit alpha(PCCA)
P05166	propionyl-CoA carboxylase subunit beta(PCCB)
P05386	ribosomal protein lateral stalk subunit P1(RPLP1)

P05387	ribosomal protein lateral stalk subunit P2(RPLP2)
P05388	ribosomal protein lateral stalk subunit P0(RPLP0)
P05451	regenerating family member 1 alpha(REG1A)
P05455	small RNA binding exonuclease protection factor La(SSB)
P05556	integrin subunit beta 1(ITGB1)
P05783	keratin 18(KRT18)
P05787	keratin 8(KRT8)
P06396	gelsolin(GSN)
P06454	prothymosin alpha(PTMA)
P06576	ATP synthase F1 subunit beta(ATP5F1B)
P06702	S100 calcium binding protein A9(S100A9)
P06703	S100 calcium binding protein A6(S100A6)
P06730	eukaryotic translation initiation factor 4E(EIF4E)
P06733	enolase 1(ENO1)
P06744	glucose-6-phosphate isomerase(GPI)
P06748	nucleophosmin 1(NPM1)
P06753	tropomyosin 3(TPM3)
P06865	hexosaminidase subunit alpha(HEXA)
P06899	H2B clustered histone 11(H2BC11)
P07108	diazepam binding inhibitor, acyl-CoA binding protein(DBI)
P07148	fatty acid binding protein 1(FABP1)
P07195	lactate dehydrogenase B(LDHB)
P07205	phosphoglycerate kinase 2(PGK2)
P07237	prolyl 4-hydroxylase subunit beta(P4HB)
P07305	H1.0 linker histone(H1-0)
P07327	alcohol dehydrogenase 1A (class I), alpha polypeptide(ADH1A)
P07339	cathepsin D(CTSD)
P07355	annexin A2(ANXA2)

P07384	calpain 1(CAPN1)
P07437	tubulin beta class I(TUBB)
P07602	prosaposin(PSAP)
P07686	hexosaminidase subunit beta(HEXB)
P07737	profilin 1(PFN1)
P07741	adenine phosphoribosyltransferase(APRT)
P07814	glutamyl-prolyl-tRNA synthetase 1(EPRS1)
P07858	cathepsin B(CTSB)
P07864	lactate dehydrogenase C(LDHC)
P07900	heat shock protein 90 alpha family class A member 1(HSP90AA1)
P07910	heterogeneous nuclear ribonucleoprotein C(HNRNPC)
P07919	ubiquinol-cytochrome c reductase hinge protein(UQCRH)
P07947	YES proto-oncogene 1, Src family tyrosine kinase(YES1)
P07948	LYN proto-oncogene, Src family tyrosine kinase(LYN)
P07951	tropomyosin 2(TPM2)
P07954	fumarate hydratase(FH)
P08133	annexin A6(ANXA6)
P08134	ras homolog family member C(RHOC)
P08238	heat shock protein 90 alpha family class B member 1(HSP90AB1)
P08559	pyruvate dehydrogenase E1 subunit alpha 1(PDHA1)
P08574	cytochrome c1(CYC1)
P08670	vimentin(VIM)
P08708	ribosomal protein S17(RPS17)
P08727	keratin 19(KRT19)
P08754	G protein subunit alpha i3(GNAI3)
P08758	annexin A5(ANXA5)
P08779	keratin 16(KRT16)
P08861	chymotrypsin like elastase 3B(CELA3B)

P08865	ribosomal protein SA(RPSA)
P09012	small nuclear ribonucleoprotein polypeptide A(SNRPA)
P09093	chymotrypsin like elastase 3A(CELA3A)
P09104	enolase 2(ENO2)
P09110	acetyl-CoA acyltransferase 1(ACAA1)
P09211	glutathione S-transferase pi 1(GSTP1)
P09327	villin 1(VIL1)
P09382	galectin 1(LGALS1)
P09429	high mobility group box 1(HMGB1)
P09467	fructose-bisphosphatase 1(FBP1)
P09493	tropomyosin 1(TPM1)
P09496	clathrin light chain A(CLTA)
P09525	annexin A4(ANXA4)
P09622	dihydrolipoamide dehydrogenase(DLD)
P09651	heterogeneous nuclear ribonucleoprotein A1(HNRNPA1)
P09669	cytochrome c oxidase subunit 6C(COX6C)
P09874	poly(ADP-ribose) polymerase 1(PARP1)
P09960	leukotriene A4 hydrolase(LTA4H)
P09972	aldolase, fructose-bisphosphate C(ALDOC)
P0C0S5	H2A.Z variant histone 1(H2AZ1)
P0C0S8	H2A clustered histone 17(H2AC17)
P0C0S8	H2A clustered histone 13(H2AC13)
P0C0S8	H2A clustered histone 11(H2AC11)
P0C0S8	H2A clustered histone 15(H2AC15)
P0C0S8	H2A clustered histone 16(H2AC16)
P0C7P4	ubiquinol-cytochrome c reductase, Rieske iron-sulfur polypeptide 1 pseudogene 1(UQCRFS1P1)
P0CG04	immunoglobulin lambda constant 1(IGLC1)
P0CG47	ubiquitin B(UBB)

P0CG48	ubiquitin C(UBC)
P0DME0	SET like protein(SETSIP)
P10155	Ro60, Y RNA binding protein(RO60)
P10253	alpha glucosidase(GAA)
P10412	H1.4 linker histone, cluster member(H1-4)
P10515	dihydrolipoamide S-acetyltransferase(DLAT)
P10599	thioredoxin(TXN)
P10606	cytochrome c oxidase subunit 5B(COX5B)
P10645	chromogranin A(CHGA)
P10768	esterase D(ESD)
P10809	heat shock protein family D (Hsp60) member 1(HSPD1)
P11021	heat shock protein family A (Hsp70) member 5(HSPA5)
P11142	heat shock protein family A (Hsp70) member 8(HSPA8)
P11177	pyruvate dehydrogenase E1 subunit beta(PDHB)
P11216	glycogen phosphorylase B(PYGB)
P11233	RAS like proto-oncogene A(RALA)
P11234	RAS like proto-oncogene B(RALB)
P11310	acyl-CoA dehydrogenase medium chain(ACADM)
P11413	glucose-6-phosphate dehydrogenase(G6PD)
P11586	methylenetetrahydrofolate dehydrogenase, cyclohydrolase and formyltetrahydrofolate synthetase 1(MTHFD1)
P11678	eosinophil peroxidase(EPX)
P11766	alcohol dehydrogenase 5 (class III), chi polypeptide(ADH5)
P11908	phosphoribosyl pyrophosphate synthetase 2(PRPS2)
P11940	poly(A) binding protein cytoplasmic 1(PABPC1)
P12004	proliferating cell nuclear antigen(PCNA)
P12081	histidyl-tRNA synthetase 1(HARS1)
P12109	collagen type VI alpha 1 chain(COL6A1)
P12111	collagen type VI alpha 3 chain(COL6A3)

P12235	solute carrier family 25 member 4(SLC25A4)
P12236	solute carrier family 25 member 6(SLC25A6)
P12268	inosine monophosphate dehydrogenase 2(IMPDH2)
P12270	translocated promoter region, nuclear basket protein(TPR)
P12277	creatine kinase B(CKB)
P12429	annexin A3(ANXA3)
P12532	creatine kinase, mitochondrial 1B(CKMT1B)
P12532	creatine kinase, mitochondrial 1A(CKMT1A)
P12724	ribonuclease A family member 3(RNASE3)
P12814	actinin alpha 1(ACTN1)
P12830	cadherin 1(CDH1)
P12955	peptidase D(PEPD)
P12956	X-ray repair cross complementing 6(XRCC6)
P13010	X-ray repair cross complementing 5(XRCC5)
P13073	cytochrome c oxidase subunit 4I1(COX4I1)
P13284	IFI30 lysosomal thiol reductase(IFI30)
P13473	lysosomal associated membrane protein 2(LAMP2)
P13489	ribonuclease/angiogenin inhibitor 1(RNH1)
P13611	versican(VCAN)
P13639	eukaryotic translation elongation factor 2(EEF2)
P13640	metallothionein 1G(MT1G)
P13645	keratin 10(KRT10)
P13647	keratin 5(KRT5)
P13667	protein disulfide isomerase family A member 4(PDIA4)
P13693	tumor protein, translationally-controlled 1(TPT1)
P13796	lymphocyte cytosolic protein 1(LCP1)
P13798	acylaminoacyl-peptide hydrolase(APEH)
P13804	electron transfer flavoprotein subunit alpha(ETFA)

P13861	protein kinase cAMP-dependent type II regulatory subunit alpha(PRKAR2A)
P13929	enolase 3(ENO3)
P13987	CD59 molecule (CD59 blood group)(CD59)
P14174	macrophage migration inhibitory factor(MIF)
P14209	CD99 molecule (Xg blood group)(CD99)
P14314	protein kinase C substrate 80K-H(PRKCSH)
P14406	cytochrome c oxidase subunit 7A2(COX7A2)
P14550	aldo-keto reductase family 1 member A1(AKR1A1)
P14618	pyruvate kinase M1/2(PKM)
P14625	heat shock protein 90 beta family member 1(HSP90B1)
P14678	small nuclear ribonucleoprotein polypeptides B and B1(SNRPB)
P14854	cytochrome c oxidase subunit 6B1(COX6B1)
P14866	heterogeneous nuclear ribonucleoprotein L(HNRNPL)
P14868	aspartyl-tRNA synthetase 1(DARS1)
P14923	junction plakoglobin(JUP)
P15121	aldo-keto reductase family 1 member B(AKR1B1)
P15153	Rac family small GTPase 2(RAC2)
P15259	phosphoglycerate mutase 2(PGAM2)
P15289	arylsulfatase A(ARSA)
P15311	ezrin(EZR)
P15374	ubiquitin C-terminal hydrolase L3(UCHL3)
P15428	15-hydroxyprostaglandin dehydrogenase(HPGD)
P15531	NME/NM23 nucleoside diphosphate kinase 1(NME1)
P15880	ribosomal protein S2(RPS2)
P15924	desmoplakin(DSP)
P16070	CD44 molecule (Indian blood group)(CD44)
P16104	H2A.X variant histone(H2AX)
P16144	integrin subunit beta 4(ITGB4)

P16152	carbonyl reductase 1(CBR1)
P16219	acyl-CoA dehydrogenase short chain(ACADS)
P16278	galactosidase beta 1(GLB1)
P16401	H1.5 linker histone, cluster member(H1-5)
P16402	H1.3 linker histone, cluster member(H1-3)
P16403	H1.2 linker histone, cluster member(H1-2)
P16422	epithelial cell adhesion molecule(EPCAM)
P16615	ATPase sarcoplasmic/endoplasmic reticulum Ca2+ transporting 2(ATP2A2)
P16949	stathmin 1(STMN1)
P17066	heat shock protein family A (Hsp70) member 6(HSPA6)
P17081	ras homolog family member Q(RHOQ)
P17174	glutamic-oxaloacetic transaminase 1(GOT1)
P17516	aldo-keto reductase family 1 member C4(AKR1C4)
P17655	calpain 2(CAPN2)
P17661	desmin(DES)
P17844	DEAD-box helicase 5(DDX5)
P17858	phosphofructokinase, liver type(PFKL)
P17931	galectin 3(LGALS3)
P17980	proteasome 26S subunit, ATPase 3(PSMC3)
P17987	t-complex 1(TCP1)
P18065	insulin like growth factor binding protein 2(IGFBP2)
P18085	ADP ribosylation factor 4(ARF4)
P18124	ribosomal protein L7(RPL7)
P18206	vinculin(VCL)
P18283	glutathione peroxidase 2(GPX2)
P18669	phosphoglycerate mutase 1(PGAM1)
P19012	keratin 15(KRT15)
P19075	tetraspanin 8(TSPAN8)

P19224	UDP glucuronosyltransferase family 1 member A6(UGT1A6)
P19338	nucleolin(NCL)
P19367	hexokinase 1(HK1)
P19404	NADH:ubiquinone oxidoreductase core subunit V2(NDUFV2)
P19801	amine oxidase copper containing 1(AOC1)
P19827	inter-alpha-trypsin inhibitor heavy chain 1(ITIH1)
P19961	amylase alpha 2B(AMY2B)
P19971	thymidine phosphorylase(TYMP)
P20073	annexin A7(ANXA7)
P20231	tryptase beta 2(TPSB2)
P20339	RAB5A, member RAS oncogene family(RAB5A)
P20340	RAB6A, member RAS oncogene family(RAB6A)
P20618	proteasome 20S subunit beta 1(PSMB1)
P20648	ATPase H+/K+ transporting subunit alpha(ATP4A)
P20671	H2A clustered histone 7(H2AC7)
P20674	cytochrome c oxidase subunit 5A(COX5A)
P20700	lamin B1(LMNB1)
P20774	osteoglycin(OGN)
P20794	male germ cell associated kinase(MAK)
P20810	calpastatin(CAST)
P20933	aspartylglucosaminidase(AGA)
P20962	parathymosin(PTMS)
P21266	glutathione S-transferase mu 3(GSTM3)
P21281	ATPase H+ transporting V1 subunit B2(ATP6V1B2)
P21291	cysteine and glycine rich protein 1(CSRP1)
P21333	filamin A(FLNA)
P21397	monoamine oxidase A(MAOA)
P21399	aconitase 1(ACO1)

P21796	voltage dependent anion channel 1(VDAC1)
P21926	CD9 molecule(CD9)
P21964	catechol-O-methyltransferase(COMT)
P21980	transglutaminase 2(TGM2)
P22102	phosphoribosylglycinamide formyltransferase, phosphoribosylglycinamide synthetase, phosphoribosylaminoimidazole synthetase(GART)
P22234	phosphoribosylaminoimidazole carboxylase and phosphoribosylaminoimidazolesuccinocarboxamide synthase(PAICS)
P22307	sterol carrier protein 2(SCP2)
P22309	UDP glucuronosyltransferase family 1 member A1(UGT1A1)
P22310	UDP glucuronosyltransferase family 1 member A4(UGT1A4)
P22314	ubiquitin like modifier activating enzyme 1(UBA1)
P22392	NME/NM23 nucleoside diphosphate kinase 2(NME2)
P22492	H1.6 linker histone, cluster member(H1-6)
P22607	fibroblast growth factor receptor 3(FGFR3)
P22626	heterogeneous nuclear ribonucleoprotein A2/B1(HNRNPA2B1)
P22695	ubiquinol-cytochrome c reductase core protein 2(UQCRC2)
P23229	integrin subunit alpha 6(ITGA6)
P23246	splicing factor proline and glutamine rich(SFPQ)
P23284	peptidylprolyl isomerase B(PPIB)
P23368	malic enzyme 2(ME2)
P23381	tryptophanyl-tRNA synthetase 1(WARS1)
P23396	ribosomal protein S3(RPS3)
P23526	adenosylhomocysteinase(AHCY)
P23527	H2B clustered histone 17(H2BC17)
P23528	cofilin 1(CFL1)
P23786	carnitine palmitoyltransferase 2(CPT2)
P24298	glutamic--pyruvic transaminase(GPT)
P24534	eukaryotic translation elongation factor 1 beta 2(EEF1B2)

P24539	ATP synthase peripheral stalk-membrane subunit b(ATP5PB)
P24752	acetyl-CoA acetyltransferase 1(ACAT1)
P24941	cyclin dependent kinase 2(CDK2)
P25325	mercaptopyruvate sulfurtransferase(MPST)
P25398	ribosomal protein S12(RPS12)
P25685	DnaJ heat shock protein family (Hsp40) member B1(DNAJB1)
P25705	ATP synthase F1 subunit alpha(ATP5F1A)
P25774	cathepsin S(CTSS)
P25786	proteasome 20S subunit alpha 1(PSMA1)
P25787	proteasome 20S subunit alpha 2(PSMA2)
P25788	proteasome 20S subunit alpha 3(PSMA3)
P25789	proteasome 20S subunit alpha 4(PSMA4)
P25815	S100 calcium binding protein P(S100P)
P26038	moesin(MSN)
P26232	catenin alpha 2(CTNNA2)
P26373	ribosomal protein L13(RPL13)
P26440	isovaleryl-CoA dehydrogenase(IVD)
P26447	S100 calcium binding protein A4(S100A4)
P26583	high mobility group box 2(HMGB2)
P26599	polypyrimidine tract binding protein 1(PTBP1)
P26639	threonyl-tRNA synthetase 1(TARS1)
P26640	valyl-tRNA synthetase 1(VARS1)
P26641	eukaryotic translation elongation factor 1 gamma(EEF1G)
P26885	FKBP prolyl isomerase 2(FKBP2)
P27105	stomatin(STOM)
P27216	annexin A13(ANXA13)
P27348	tyrosine 3-monooxygenase/tryptophan 5-monooxygenase activation protein theta(YWHAQ)
P27635	ribosomal protein L10(RPL10)

P27695	apurinic/apyrimidinic endodeoxyribonuclease 1(APEX1)
P27797	calreticulin(CALR)
P27816	microtubule associated protein 4(MAP4)
P27824	calnexin(CANX)
P28062	proteasome 20S subunit beta 8(PSMB8)
P28065	proteasome 20S subunit beta 9(PSMB9)
P28066	proteasome 20S subunit alpha 5(PSMA5)
P28070	proteasome 20S subunit beta 4(PSMB4)
P28072	proteasome 20S subunit beta 6(PSMB6)
P28161	glutathione S-transferase mu 2(GSTM2)
P28331	NADH:ubiquinone oxidoreductase core subunit S1(NDUFS1)
P28482	mitogen-activated protein kinase 1(MAPK1)
P28799	granulin precursor(GRN)
P28838	leucine aminopeptidase 3(LAP3)
P29401	transketolase(TKT)
P29466	caspase 1(CASP1)
P29692	eukaryotic translation elongation factor 1 delta(EEF1D)
P29966	myristoylated alanine rich protein kinase C substrate(MARCKS)
P30039	phenazine biosynthesis like protein domain containing(PBLD)
P30040	endoplasmic reticulum protein 29(ERP29)
P30041	peroxiredoxin 6(PRDX6)
P30043	biliverdin reductase B(BLVRB)
P30044	peroxiredoxin 5(PRDX5)
P30046	D-dopachrome tautomerase(DDT)
P30048	peroxiredoxin 3(PRDX3)
P30049	ATP synthase F1 subunit delta(ATP5F1D)
P30050	ribosomal protein L12(RPL12)
P30084	enoyl-CoA hydratase, short chain 1(ECHS1)

P30085	cytidine/uridine monophosphate kinase 1(CMPK1)
P30086	phosphatidylethanolamine binding protein 1(PEBP1)
P30101	protein disulfide isomerase family A member 3(PDIA3)
P30153	protein phosphatase 2 scaffold subunit Aalpha(PPP2R1A)
P30419	N-myristoyltransferase 1(NMT1)
P30626	sorcin(SRI)
P30740	serpin family B member 1(SERPINB1)
P30837	aldehyde dehydrogenase 1 family member B1(ALDH1B1)
P31040	succinate dehydrogenase complex flavoprotein subunit A(SDHA)
P31146	coronin 1A(CORO1A)
P31150	GDP dissociation inhibitor 1(GDI1)
P31153	methionine adenosyltransferase 2A(MAT2A)
P31930	ubiquinol-cytochrome c reductase core protein 1(UQCRC1)
P31937	3-hydroxyisobutyrate dehydrogenase(HIBADH)
P31939	5-aminoimidazole-4-carboxamide ribonucleotide formyltransferase/IMP cyclohydrolase(ATIC)
P31942	heterogeneous nuclear ribonucleoprotein H3(HNRNPH3)
P31943	heterogeneous nuclear ribonucleoprotein H1(HNRNPH1)
P31946	tyrosine 3-monooxygenase/tryptophan 5-monooxygenase activation protein beta(YWHAB)
P31947	stratifin(SFN)
P31948	stress induced phosphoprotein 1(STIP1)
P31949	S100 calcium binding protein A11(S100A11)
P32119	peroxiredoxin 2(PRDX2)
P32455	guanylate binding protein 1(GBP1)
P33176	kinesin family member 5B(KIF5B)
P33240	cleavage stimulation factor subunit 2(CSTF2)
P33316	deoxyuridine triphosphatase(DUT)
P33778	H2B clustered histone 3(H2BC3)

P34897	serine hydroxymethyltransferase 2(SHMT2)
P34932	heat shock protein family A (Hsp70) member 4(HSPA4)
P35080	profilin 2(PFN2)
P35221	catenin alpha 1(CTNNA1)
P35222	catenin beta 1(CTNNB1)
P35232	prohibitin 1(PHB1)
P35237	serpin family B member 6(SERPINB6)
P35268	ribosomal protein L22(RPL22)
P35270	sepiapterin reductase(SPR)
P35503	UDP glucuronosyltransferase family 1 member A3(UGT1A3)
P35504	UDP glucuronosyltransferase family 1 member A5(UGT1A5)
P35527	keratin 9(KRT9)
P35555	fibrillin 1(FBN1)
P35579	myosin heavy chain 9(MYH9)
P35606	COPI coat complex subunit beta 2(COPB2)
P35611	adducin 1(ADD1)
P35613	basigin (Ok blood group)(BSG)
P35637	FUS RNA binding protein(FUS)
P35659	DEK proto-oncogene(DEK)
P35749	myosin heavy chain 11(MYH11)
P35900	keratin 20(KRT20)
P35908	keratin 2(KRT2)
P35914	3-hydroxy-3-methylglutaryl-CoA lyase(HMGCL)
P35998	proteasome 26S subunit, ATPase 2(PSMC2)
P36269	gamma-glutamyltransferase 5(GGT5)
P36542	ATP synthase F1 subunit gamma(ATP5F1C)
P36578	ribosomal protein L4(RPL4)
P36871	phosphoglucomutase 1(PGM1)

P36873	protein phosphatase 1 catalytic subunit gamma(PPP1CC)
P36957	dihydrolipoamide S-succinyltransferase(DLST)
P37059	hydroxysteroid 17-beta dehydrogenase 2(HSD17B2)
P37802	transgelin 2(TAGLN2)
P37837	transaldolase 1(TALDO1)
P38117	electron transfer flavoprotein subunit beta(ETFB)
P38159	RNA binding motif protein X-linked(RBMX)
P38606	ATPase H+ transporting V1 subunit A(ATP6V1A)
P38646	heat shock protein family A (Hsp70) member 9(HSPA9)
P38919	eukaryotic translation initiation factor 4A3(EIF4A3)
P39019	ribosomal protein S19(RPS19)
P39023	ribosomal protein L3(RPL3)
P39656	dolichyl-diphosphooligosaccharide--protein glycosyltransferase non-catalytic subunit(DDOST)
P39687	acidic nuclear phosphoprotein 32 family member A(ANP32A)
P40121	capping actin protein, gelsolin like(CAPG)
P40227	chaperonin containing TCP1 subunit 6A(CCT6A)
P40306	proteasome 20S subunit beta 10(PSMB10)
P40429	ribosomal protein L13a(RPL13A)
P40616	ADP ribosylation factor like GTPase 1(ARL1)
P40763	signal transducer and activator of transcription 3(STAT3)
P40925	malate dehydrogenase 1(MDH1)
P40926	malate dehydrogenase 2(MDH2)
P40939	hydroxyacyl-CoA dehydrogenase trifunctional multienzyme complex subunit alpha(HADHA)
P41091	eukaryotic translation initiation factor 2 subunit gamma(EIF2S3)
P41250	glycyl-tRNA synthetase 1(GARS1)
P42126	enoyl-CoA delta isomerase 1(ECI1)
P42166	thymopoietin(TMPO)

P42167	thymopoietin(TMPO)
P42224	signal transducer and activator of transcription 1(STAT1)
P42330	aldo-keto reductase family 1 member C3(AKR1C3)
P42574	caspase 3(CASP3)
P42677	ribosomal protein S27(RPS27)
P42704	leucine rich pentatricopeptide repeat containing(LRPPRC)
P42765	acetyl-CoA acyltransferase 2(ACAA2)
P43034	platelet activating factor acetylhydrolase 1b regulatory subunit 1(PAFAH1B1)
P43121	melanoma cell adhesion molecule(MCAM)
P43155	carnitine O-acetyltransferase(CRAT)
P43243	matrin 3(MATR3)
P43304	glycerol-3-phosphate dehydrogenase 2(GPD2)
P43307	signal sequence receptor subunit 1(SSR1)
P43487	RAN binding protein 1(RANBP1)
P43490	nicotinamide phosphoribosyltransferase(NAMPT)
P43686	proteasome 26S subunit, ATPase 4(PSMC4)
P43897	Ts translation elongation factor, mitochondrial(TSFM)
P45880	voltage dependent anion channel 2(VDAC2)
P45954	acyl-CoA dehydrogenase short/branched chain(ACADSB)
P45974	ubiquitin specific peptidase 5(USP5)
P46439	glutathione S-transferase mu 5(GSTM5)
P46459	N-ethylmaleimide sensitive factor, vesicle fusing ATPase(NSF)
P46776	ribosomal protein L27a(RPL27A)
P46777	ribosomal protein L5(RPL5)
P46782	ribosomal protein S5(RPS5)
P46940	IQ motif containing GTPase activating protein 1(IQGAP1)
P46977	STT3 oligosaccharyltransferase complex catalytic subunit A(STT3A)
P47755	capping actin protein of muscle Z-line subunit alpha 2(CAPZA2)

P47756	capping actin protein of muscle Z-line subunit beta(CAPZB)
P47897	glutaminyl-tRNA synthetase 1(QARS1)
P47914	ribosomal protein L29(RPL29)
P47985	ubiquinol-cytochrome c reductase, Rieske iron-sulfur polypeptide 1(UQCRFS1)
P48047	ATP synthase peripheral stalk subunit OSCP(ATP5PO)
P48147	prolyl endopeptidase(PREP)
P48304	regenerating family member 1 beta(REG1B)
P48444	archain 1(ARCN1)
P48509	CD151 molecule (Raph blood group)(CD151)
P48637	glutathione synthetase(GSS)
P48643	chaperonin containing TCP1 subunit 5(CCT5)
P48668	keratin 6C(KRT6C)
P48735	isocitrate dehydrogenase (NADP(+)) 2(IDH2)
P49006	MARCKS like 1(MARCKSL1)
P49189	aldehyde dehydrogenase 9 family member A1(ALDH9A1)
P49207	ribosomal protein L34(RPL34)
P49257	lectin, mannose binding 1(LMAN1)
P49321	nuclear autoantigenic sperm protein(NASP)
P49327	fatty acid synthase(FASN)
P49368	chaperonin containing TCP1 subunit 3(CCT3)
P49411	Tu translation elongation factor, mitochondrial(TUFM)
P49419	aldehyde dehydrogenase 7 family member A1(ALDH7A1)
P49448	glutamate dehydrogenase 2(GLUD2)
P49458	signal recognition particle 9(SRP9)
P49588	alanyl-tRNA synthetase 1(AARS1)
P49590	histidyl-tRNA synthetase 2, mitochondrial(HARS2)
P49591	seryl-tRNA synthetase 1(SARS1)
P49720	proteasome 20S subunit beta 3(PSMB3)

P49748	acyl-CoA dehydrogenase very long chain(ACADVL)
P49753	acyl-CoA thioesterase 2(ACOT2)
P49755	transmembrane p24 trafficking protein 10(TMED10)
P49756	RNA binding motif protein 25(RBM25)
P49821	NADH:ubiquinone oxidoreductase core subunit V1(NDUFV1)
P50135	histamine N-methyltransferase(HNMT)
P50213	isocitrate dehydrogenase (NAD(+)) 3 catalytic subunit alpha(IDH3A)
P50395	GDP dissociation inhibitor 2(GDI2)
P50416	carnitine palmitoyltransferase 1A(CPT1A)
P50454	serpin family H member 1(SERPINH1)
P50502	ST13 Hsp70 interacting protein(ST13)
P50552	vasodilator stimulated phosphoprotein(VASP)
P50570	dynamin 2(DNM2)
P50897	palmitoyl-protein thioesterase 1(PPT1)
P50914	ribosomal protein L14(RPL14)
P50990	chaperonin containing TCP1 subunit 8(CCT8)
P50991	chaperonin containing TCP1 subunit 4(CCT4)
P50993	ATPase Na+/K+ transporting subunit alpha 2(ATP1A2)
P50995	annexin A11(ANXA11)
P51148	RAB5C, member RAS oncogene family(RAB5C)
P51149	RAB7A, member RAS oncogene family(RAB7A)
P51159	RAB27A, member RAS oncogene family(RAB27A)
P51572	B cell receptor associated protein 31(BCAP31)
P51648	aldehyde dehydrogenase 3 family member A2(ALDH3A2)
P51659	hydroxysteroid 17-beta dehydrogenase 4(HSD17B4)
P51665	proteasome 26S subunit, non-ATPase 7(PSMD7)
P51812	ribosomal protein S6 kinase A3(RPS6KA3)
P51858	heparin binding growth factor(HDGF)

P51884	lumican(LUM)
P51991	heterogeneous nuclear ribonucleoprotein A3(HNRNPA3)
P52209	phosphogluconate dehydrogenase(PGD)
P52272	heterogeneous nuclear ribonucleoprotein M(HNRNPM)
P52306	Rap1 GTPase-GDP dissociation stimulator 1(RAP1GDS1)
P52565	Rho GDP dissociation inhibitor alpha(ARHGDIA)
P52566	Rho GDP dissociation inhibitor beta(ARHGDIB)
P52597	heterogeneous nuclear ribonucleoprotein F(HNRNPF)
P52789	hexokinase 2(HK2)
P52815	mitochondrial ribosomal protein L12(MRPL12)
P52895	aldo-keto reductase family 1 member C2(AKR1C2)
P52907	capping actin protein of muscle Z-line subunit alpha 1(CAPZA1)
P53367	ADP ribosylation factor interacting protein 1(ARFIP1)
P53597	succinate-CoA ligase GDP/ADP-forming subunit alpha(SUCLG1)
P53618	COPI coat complex subunit beta 1(COPB1)
P53621	COPI coat complex subunit alpha(COPA)
P53634	cathepsin C(CTSC)
P54136	arginyl-tRNA synthetase 1(RARS1)
P54652	heat shock protein family A (Hsp70) member 2(HSPA2)
P54727	RAD23 homolog B, nucleotide excision repair protein(RAD23B)
P54819	adenylate kinase 2(AK2)
P54868	3-hydroxy-3-methylglutaryl-CoA synthase 2(HMGCS2)
P54886	aldehyde dehydrogenase 18 family member A1(ALDH18A1)
P55010	eukaryotic translation initiation factor 5(EIF5)
P55011	solute carrier family 12 member 2(SLC12A2)
P55072	valosin containing protein(VCP)
P55084	hydroxyacyl-CoA dehydrogenase trifunctional multienzyme complex subunit beta(HADHB)
P55145	mesencephalic astrocyte derived neurotrophic factor(MANF)

P55209	nucleosome assembly protein 1 like 1(NAP1L1)
P55265	adenosine deaminase RNA specific(ADAR)
P55290	cadherin 13(CDH13)
P55327	tumor protein D52(TPD52)
P55769	small nuclear ribonucleoprotein 13(SNU13)
P55786	aminopeptidase puromycin sensitive(NPEPPS)
P55795	heterogeneous nuclear ribonucleoprotein H2(HNRNPH2)
P55809	3-oxoacid CoA-transferase 1(OXCT1)
P55854	small ubiquitin like modifier 3(SUMO3)
P55884	eukaryotic translation initiation factor 3 subunit B(EIF3B)
P56134	ATP synthase membrane subunit f(ATP5MF)
P56199	integrin subunit alpha 1(ITGA1)
P56385	ATP synthase membrane subunit e(ATP5ME)
P56470	galectin 4(LGALS4)
P56537	eukaryotic translation initiation factor 6(EIF6)
P56545	C-terminal binding protein 2(CTBP2)
P57053	H2B clustered histone 12 like(H2BC12L)
P57088	transmembrane protein 33(TMEM33)
P57737	coronin 7(CORO7)
P58107	epiplakin 1(EPPK1)
P58335	ANTXR cell adhesion molecule 2(ANTXR2)
P58546	myotrophin(MTPN)
P58876	H2B clustered histone 5(H2BC5)
P59665	defensin alpha 1(DEFA1)
P59665	defensin alpha 1B(DEFA1B)
P59666	defensin alpha 3(DEFA3)
P59998	actin related protein 2/3 complex subunit 4(ARPC4)
P60033	CD81 molecule(CD81)

P60174	triosephosphate isomerase 1(TPI1)
P60228	eukaryotic translation initiation factor 3 subunit E(EIF3E)
P60468	SEC61 translocon subunit beta(SEC61B)
P60660	myosin light chain 6(MYL6)
P60709	actin beta(ACTB)
P60712	actin beta(ACTB)
P60763	Rac family small GTPase 3(RAC3)
P60842	eukaryotic translation initiation factor 4A1(EIF4A1)
P60866	ribosomal protein S20(RPS20)
P60891	phosphoribosyl pyrophosphate synthetase 1(PRPS1)
P60900	proteasome 20S subunit alpha 6(PSMA6)
P60903	S100 calcium binding protein A10(S100A10)
P60953	cell division cycle 42(CDC42)
P60981	destrin, actin depolymerizing factor(DSTN)
P60983	glia maturation factor beta(GMFB)
P61006	RAB8A, member RAS oncogene family(RAB8A)
P61019	RAB2A, member RAS oncogene family(RAB2A)
P61026	RAB10, member RAS oncogene family(RAB10)
P61088	ubiquitin conjugating enzyme E2 N(UBE2N)
P61106	RAB14, member RAS oncogene family(RAB14)
P61158	actin related protein 3(ACTR3)
P61160	actin related protein 2(ACTR2)
P61204	ADP ribosylation factor 3(ARF3)
P61221	ATP binding cassette subfamily E member 1(ABCE1)
P61224	RAP1B, member of RAS oncogene family(RAP1B)
P61247	ribosomal protein S3A(RPS3A)
P61254	ribosomal protein L26(RPL26)
P61313	ribosomal protein L15(RPL15)

P61513	ribosomal protein L37a(RPL37A)
P61586	ras homolog family member A(RHOA)
P61604	heat shock protein family E (Hsp10) member 1(HSPE1)
P61619	SEC61 translocon subunit alpha 1(SEC61A1)
P61769	beta-2-microglobulin(B2M)
P61803	defender against cell death 1(DAD1)
P61916	NPC intracellular cholesterol transporter 2(NPC2)
P61956	small ubiquitin like modifier 2(SUMO2)
P61978	heterogeneous nuclear ribonucleoprotein K(HNRNPK)
P61981	tyrosine 3-monooxygenase/tryptophan 5-monooxygenase activation protein gamma(YWHAG)
P62081	ribosomal protein S7(RPS7)
P62136	protein phosphatase 1 catalytic subunit alpha(PPP1CA)
P62140	protein phosphatase 1 catalytic subunit beta(PPP1CB)
P62191	proteasome 26S subunit, ATPase 1(PSMC1)
P62195	proteasome 26S subunit, ATPase 5(PSMC5)
P62241	ribosomal protein S8(RPS8)
P62244	ribosomal protein S15a(RPS15A)
P62249	ribosomal protein S16(RPS16)
P62258	tyrosine 3-monooxygenase/tryptophan 5-monooxygenase activation protein epsilon(YWHAE)
P62263	ribosomal protein S14(RPS14)
P62266	ribosomal protein S23(RPS23)
P62269	ribosomal protein S18(RPS18)
P62277	ribosomal protein S13(RPS13)
P62280	ribosomal protein S11(RPS11)
P62306	small nuclear ribonucleoprotein polypeptide F(SNRPF)
P62308	small nuclear ribonucleoprotein polypeptide G(SNRPG)
P62316	small nuclear ribonucleoprotein D2 polypeptide(SNRPD2)

P62318	small nuclear ribonucleoprotein D3 polypeptide(SNRPD3)
P62328	thymosin beta 4 X-linked(TMSB4X)
P62333	proteasome 26S subunit, ATPase 6(PSMC6)
P62424	ribosomal protein L7a(RPL7A)
P62491	RAB11A, member RAS oncogene family(RAB11A)
P62633	CCHC-type zinc finger nucleic acid binding protein(CNBP)
P62701	ribosomal protein S4 X-linked(RPS4X)
P62736	actin alpha 2, smooth muscle(ACTA2)
P62750	ribosomal protein L23a(RPL23A)
P62753	ribosomal protein S6(RPS6)
P62805	H4 histone 16(H4C16)
P62805	H4 clustered histone 15(H4C15)
P62805	H4 clustered histone 9(H4C9)
P62805	H4 clustered histone 1(H4C1)
P62805	H4 clustered histone 4(H4C4)
P62805	H4 clustered histone 6(H4C6)
P62805	H4 clustered histone 12(H4C12)
P62805	H4 clustered histone 11(H4C11)
P62805	H4 clustered histone 3(H4C3)
P62805	H4 clustered histone 8(H4C8)
P62805	H4 clustered histone 2(H4C2)
P62805	H4 clustered histone 5(H4C5)
P62805	H4 clustered histone 13(H4C13)
P62805	H4 clustered histone 14(H4C14)
P62807	H2B clustered histone 8(H2BC8)
P62807	H2B clustered histone 7(H2BC7)
P62807	H2B clustered histone 6(H2BC6)
P62807	H2B clustered histone 10(H2BC10)

P62807	H2B clustered histone 4(H2BC4)
P62820	RAB1A, member RAS oncogene family(RAB1A)
P62826	RAN, member RAS oncogene family(RAN)
P62829	ribosomal protein L23(RPL23)
P62834	RAP1A, member of RAS oncogene family(RAP1A)
P62841	ribosomal protein S15(RPS15)
P62847	ribosomal protein S24(RPS24)
P62851	ribosomal protein S25(RPS25)
P62854	ribosomal protein S26(RPS26)
P62857	ribosomal protein S28(RPS28)
P62873	G protein subunit beta 1(GNB1)
P62879	G protein subunit beta 2(GNB2)
P62888	ribosomal protein L30(RPL30)
P62899	ribosomal protein L31(RPL31)
P62906	ribosomal protein L10a(RPL10A)
P62913	ribosomal protein L11(RPL11)
P62917	ribosomal protein L8(RPL8)
P62937	peptidylprolyl isomerase A(PPIA)
P62979	ribosomal protein S27a(RPS27A)
P62987	ubiquitin A-52 residue ribosomal protein fusion product 1(UBA52)
P62995	transformer 2 beta homolog(TRA2B)
P63000	Rac family small GTPase 1(RAC1)
P63027	vesicle associated membrane protein 2(VAMP2)
P63104	tyrosine 3-monooxygenase/tryptophan 5-monooxygenase activation protein zeta(YWHAZ)
P63151	protein phosphatase 2 regulatory subunit Balpha(PPP2R2A)
P63162	small nuclear ribonucleoprotein polypeptide N(SNRPN)
P63173	ribosomal protein L38(RPL38)
P63220	ribosomal protein S21(RPS21)

P63241	eukaryotic translation initiation factor 5A(EIF5A)
P63244	receptor for activated C kinase 1(RACK1)
P63261	actin gamma 1(ACTG1)
P63267	actin gamma 2, smooth muscle(ACTG2)
P63313	thymosin beta 10(TMSB10)
P67936	tropomyosin 4(TPM4)
P67983	metallothionein-1A(MT1A)
P68032	actin alpha cardiac muscle 1(ACTC1)
P68104	eukaryotic translation elongation factor 1 alpha 1(EEF1A1)
P68133	actin alpha 1, skeletal muscle(ACTA1)
P68363	tubulin alpha 1b(TUBA1B)
P68366	tubulin alpha 4a(TUBA4A)
P68371	tubulin beta 4B class IVb(TUBB4B)
P68402	platelet activating factor acetylhydrolase 1b catalytic subunit 2(PAFAH1B2)
P68431	H3 clustered histone 3(H3C3)
P68431	H3 clustered histone 6(H3C6)
P68431	H3 clustered histone 11(H3C11)
P68431	H3 clustered histone 8(H3C8)
P68431	H3 clustered histone 12(H3C12)
P68431	H3 clustered histone 10(H3C10)
P68431	H3 clustered histone 2(H3C2)
P68431	H3 clustered histone 7(H3C7)
P68431	H3 clustered histone 1(H3C1)
P68431	H3 clustered histone 4(H3C4)
P68871	hemoglobin subunit beta(HBB)
P69905	hemoglobin subunit alpha 2(HBA2)
P69905	hemoglobin subunit alpha 1(HBA1)
P78371	chaperonin containing TCP1 subunit 2(CCT2)

P78417	glutathione S-transferase omega 1(GSTO1)
P78527	protein kinase, DNA-activated, catalytic subunit(PRKDC)
P78563	adenosine deaminase RNA specific B1(ADARB1)
P80297	metallothionein 1X(MT1X)
P80303	nucleobindin 2(NUCB2)
P80365	hydroxysteroid 11-beta dehydrogenase 2(HSD11B2)
P80723	brain abundant membrane attached signal protein 1(BASP1)
P80748	immunoglobulin lambda variable 3-21(IGLV3-21)
P82979	SAP domain containing ribonucleoprotein(SARNP)
P83731	ribosomal protein L24(RPL24)
P83881	ribosomal protein L36a(RPL36A)
P84077	ADP ribosylation factor 1(ARF1)
P84085	ADP ribosylation factor 5(ARF5)
P84090	ERH mRNA splicing and mitosis factor(ERH)
P84098	ribosomal protein L19(RPL19)
P84103	serine and arginine rich splicing factor 3(SRSF3)
P84243	H3.3 histone A(H3-3A)
P84243	H3.3 histone B(H3-3B)
P99999	cytochrome c, somatic(CYCS)
Q00005	protein phosphatase 2 regulatory subunit Bbeta(PPP2R2B)
Q00059	transcription factor A, mitochondrial(TFAM)
Q00266	methionine adenosyltransferase 1A(MAT1A)
Q00325	solute carrier family 25 member 3(SLC25A3)
Q00341	high density lipoprotein binding protein(HDLBP)
Q00610	clathrin heavy chain(CLTC)
Q00765	receptor accessory protein 5(REEP5)
Q00796	sorbitol dehydrogenase(SORD)
Q00839	heterogeneous nuclear ribonucleoprotein U(HNRNPU)

Q01081	U2 small nuclear RNA auxiliary factor 1(U2AF1)
Q01082	spectrin beta, non-erythrocytic 1(SPTBN1)
Q01105	SET nuclear proto-oncogene(SET)
Q01130	serine and arginine rich splicing factor 2(SRSF2)
Q01469	fatty acid binding protein 5(FABP5)
Q01518	cyclase associated actin cytoskeleton regulatory protein 1(CAP1)
Q01813	phosphofructokinase, platelet(PFKP)
Q01814	ATPase plasma membrane Ca2+ transporting 2(ATP2B2)
Q01995	transgelin(TAGLN)
Q02218	oxoglutarate dehydrogenase(OGDH)
Q02252	aldehyde dehydrogenase 6 family member A1(ALDH6A1)
Q02338	3-hydroxybutyrate dehydrogenase 1(BDH1)
Q02388	collagen type VII alpha 1 chain(COL7A1)
Q02539	H1.1 linker histone, cluster member(H1-1)
Q02543	ribosomal protein L18a(RPL18A)
Q02750	mitogen-activated protein kinase kinase 1(MAP2K1)
Q02790	FKBP prolyl isomerase 4(FKBP4)
Q02817	mucin 2, oligomeric mucus/gel-forming(MUC2)
Q02818	nucleobindin 1(NUCB1)
Q02878	ribosomal protein L6(RPL6)
Q02978	solute carrier family 25 member 11(SLC25A11)
Q03113	G protein subunit alpha 12(GNA12)
Q03252	lamin B2(LMNB2)
Q04637	eukaryotic translation initiation factor 4 gamma 1(EIF4G1)
Q04695	keratin 17(KRT17)
Q04760	glyoxalase I(GLO1)
Q04828	aldo-keto reductase family 1 member C1(AKR1C1)
Q04837	single stranded DNA binding protein 1(SSBP1)

Q04917	tyrosine 3-monooxygenase/tryptophan 5-monooxygenase activation protein eta(YWHAH)
Q05315	Charcot-Leyden crystal galectin(CLC)
Q05682	caldesmon 1(CALD1)
Q05707	collagen type XIV alpha 1 chain(COL14A1)
Q06033	inter-alpha-trypsin inhibitor heavy chain 3(ITIH3)
Q06141	regenerating family member 3 alpha(REG3A)
Q06210	glutamine--fructose-6-phosphate transaminase 1(GFPT1)
Q06323	proteasome activator subunit 1(PSME1)
Q06830	peroxiredoxin 1(PRDX1)
Q07020	ribosomal protein L18(RPL18)
Q07021	complement C1q binding protein(C1QBP)
Q07065	cytoskeleton associated protein 4(CKAP4)
Q07654	trefoil factor 3(TFF3)
Q07955	serine and arginine rich splicing factor 1(SRSF1)
Q07960	Rho GTPase activating protein 1(ARHGAP1)
Q08211	DExH-box helicase 9(DHX9)
Q08380	galectin 3 binding protein(LGALS3BP)
Q08AM6	VAC14 component of PIKFYVE complex(VAC14)
Q09028	RB binding protein 4, chromatin remodeling factor(RBBP4)
Q09666	AHNAK nucleoprotein(AHNAK)
Q0VD83	apolipoprotein B receptor(APOBR)
Q10713	peptidase, mitochondrial processing subunit alpha(PMPCA)
Q12840	kinesin family member 5A(KIF5A)
Q12864	cadherin 17(CDH17)
Q12888	tumor protein p53 binding protein 1(TP53BP1)
Q12905	interleukin enhancer binding factor 2(ILF2)
Q12906	interleukin enhancer binding factor 3(ILF3)
Q12907	lectin, mannose binding 2(LMAN2)

Q12931	TNF receptor associated protein 1(TRAP1)
Q13011	enoyl-CoA hydratase 1(ECH1)
Q13029	PR/SET domain 2(PRDM2)
Q13075	NLR family apoptosis inhibitory protein(NAIP)
Q13148	TAR DNA binding protein(TARDBP)
Q13151	heterogeneous nuclear ribonucleoprotein A0(HNRNPA0)
Q13162	peroxiredoxin 4(PRDX4)
Q13200	proteasome 26S subunit ubiquitin receptor, non-ATPase 2(PSMD2)
Q13228	selenium binding protein 1(SELENBP1)
Q13243	serine and arginine rich splicing factor 5(SRSF5)
Q13247	serine and arginine rich splicing factor 6(SRSF6)
Q13263	tripartite motif containing 28(TRIM28)
Q13283	G3BP stress granule assembly factor 1(G3BP1)
Q13310	poly(A) binding protein cytoplasmic 4(PABPC4)
Q13363	C-terminal binding protein 1(CTBP1)
Q13404	ubiquitin conjugating enzyme E2 V1(UBE2V1)
Q13409	dynein cytoplasmic 1 intermediate chain 2(DYNC1I2)
Q13423	nicotinamide nucleotide transhydrogenase(NNT)
Q13510	N-acylsphingosine amidohydrolase 1(ASAH1)
Q13535	ATR serine/threonine kinase(ATR)
Q13547	histone deacetylase 1(HDAC1)
Q13557	calcium/calmodulin dependent protein kinase II delta(CAMK2D)
Q13630	GDP-L-fucose synthase(GFUS)
Q13642	four and a half LIM domains 1(FHL1)
Q13733	ATPase Na+/K+ transporting subunit alpha 4(ATP1A4)
Q13765	nascent polypeptide associated complex subunit alpha(NACA)
Q13813	spectrin alpha, non-erythrocytic 1(SPTAN1)
Q13838	DExD-box helicase 39B(DDX39B)

Q13885	tubulin beta 2A class IIa(TUBB2A)
Q14019	coactosin like F-actin binding protein 1(COTL1)
Q14103	heterogeneous nuclear ribonucleoprotein D(HNRNPD)
Q14126	desmoglein 2(DSG2)
Q14152	eukaryotic translation initiation factor 3 subunit A(EIF3A)
Q14165	malectin(MLEC)
Q14195	dihydropyrimidinase like 3(DPYSL3)
Q14204	dynein cytoplasmic 1 heavy chain 1(DYNC1H1)
Q14240	eukaryotic translation initiation factor 4A2(EIF4A2)
Q14247	cortactin(CTTN)
Q14254	flotillin 2(FLOT2)
Q14258	tripartite motif containing 25(TRIM25)
Q14315	filamin C(FLNC)
Q14344	G protein subunit alpha 13(GNA13)
Q14508	WAP four-disulfide core domain 2(WFDC2)
Q14651	plastin 1(PLS1)
Q14697	glucosidase II alpha subunit(GANAB)
Q14764	major vault protein(MVP)
Q14847	LIM and SH3 protein 1(LASP1)
Q14956	glycoprotein nmb(GPNMB)
Q14974	karyopherin subunit beta 1(KPNB1)
Q14980	nuclear mitotic apparatus protein 1(NUMA1)
Q149M9	NACHT and WD repeat domain containing 1(NWD1)
Q14CN4	keratin 72(KRT72)
Q15019	septin 2(SEPTIN2)
Q15029	elongation factor Tu GTP binding domain containing 2(EFTUD2)
Q15031	leucyl-tRNA synthetase 2, mitochondrial(LARS2)
Q15041	ADP ribosylation factor like GTPase 6 interacting protein 1(ARL6IP1)

Q15046	lysyl-tRNA synthetase 1(KARS1)
Q15058	kinesin family member 14(KIF14)
Q15063	periostin(POSTN)
Q15075	early endosome antigen 1(EEA1)
Q15084	protein disulfide isomerase family A member 6(PDIA6)
Q15149	plectin(PLEC)
Q15181	inorganic pyrophosphatase 1(PPA1)
Q15185	prostaglandin E synthase 3(PTGES3)
Q15233	non-POU domain containing octamer binding(NONO)
Q15274	quinolinate phosphoribosyltransferase(QPRT)
Q15293	reticulocalbin 1(RCN1)
Q15365	poly(rC) binding protein 1(PCBP1)
Q15366	poly(rC) binding protein 2(PCBP2)
Q15369	elongin C(ELOC)
Q15393	splicing factor 3b subunit 3(SF3B3)
Q15417	calponin 3(CNN3)
Q15435	protein phosphatase 1 regulatory subunit 7(PPP1R7)
Q15436	SEC23 homolog A, COPII coat complex component(SEC23A)
Q15437	SEC23 homolog B, COPII coat complex component(SEC23B)
Q15459	splicing factor 3a subunit 1(SF3A1)
Q15582	transforming growth factor beta induced(TGFBI)
Q15637	splicing factor 1(SF1)
Q15661	tryptase alpha/beta 1(TPSAB1)
Q15691	microtubule associated protein RP/EB family member 1(MAPRE1)
Q15717	ELAV like RNA binding protein 1(ELAVL1)
Q15746	myosin light chain kinase(MYLK)
Q15836	vesicle associated membrane protein 3(VAMP3)
Q15907	RAB11B, member RAS oncogene family(RAB11B)

Q16134	electron transfer flavoprotein dehydrogenase(ETFDH)
Q16181	septin 7(SEPTIN7)
Q16186	ADRM1 26S proteasome ubiquitin receptor(ADRM1)
Q16543	cell division cycle 37, HSP90 cochaperone(CDC37)
Q16555	dihydropyrimidinase like 2(DPYSL2)
Q16629	serine and arginine rich splicing factor 7(SRSF7)
Q16658	fascin actin-bundling protein 1(FSCN1)
Q16695	H3.4 histone, cluster member(H3-4)
Q16698	2,4-dienoyl-CoA reductase 1(DECR1)
Q16718	NADH:ubiquinone oxidoreductase subunit A5(NDUFA5)
Q16762	thiosulfate sulfurtransferase(TST)
Q16774	guanylate kinase 1(GUK1)
Q16777	H2A clustered histone 20(H2AC20)
Q16778	H2B clustered histone 21(H2BC21)
Q16795	NADH:ubiquinone oxidoreductase subunit A9(NDUFA9)
Q16822	phosphoenolpyruvate carboxykinase 2, mitochondrial(PCK2)
Q16836	hydroxyacyl-CoA dehydrogenase(HADH)
Q16851	UDP-glucose pyrophosphorylase 2(UGP2)
Q16853	amine oxidase copper containing 3(AOC3)
Q16881	thioredoxin reductase 1(TXNRD1)
Q16891	inner membrane mitochondrial protein(IMMT)
Q1KMD3	heterogeneous nuclear ribonucleoprotein U like 2(HNRNPUL2)
Q2NKJ3	CST telomere replication complex component 1(CTC1)
Q2VIR3	eukaryotic translation initiation factor 2 subunit gamma B(EIF2S3B)
Q32MZ4	LRR binding FLII interacting protein 1(LRRFIP1)
Q32P51	heterogeneous nuclear ribonucleoprotein A1 like 2(HNRNPA1L2)
Q3B8N2	galectin 9B(LGALS9B)
Q3KQU3	MAP7 domain containing 1(MAP7D1)

Q3LXA3	triokinase and FMN cyclase(TKFC)
Q3TTY5	keratin 2(Krt2)
Q4G176	acyl-CoA synthetase family member 3(ACSF3)
Q4V328	GRIP1 associated protein 1(GRIPAP1)
Q4VXU2	poly(A) binding protein cytoplasmic 1 like(PABPC1L)
Q53EL6	programmed cell death 4(PDCD4)
Q562R1	actin beta like 2(ACTBL2)
Q56VL3	OCIA domain containing 2(OCIAD2)
Q58FF3	heat shock protein 90 beta family member 2, pseudogene(HSP90B2P)
Q5JNZ5	ribosomal protein S26 pseudogene 11(RPS26P11)
Q5JQF8	poly(A) binding protein cytoplasmic 1 like 2A(PABPC1L2A)
Q5JQF8	poly(A) binding protein cytoplasmic 1 like 2B(PABPC1L2B)
Q5JTD0	tight junction associated protein 1(TJAP1)
Q5JXB2	ubiquitin conjugating enzyme E2 N like (gene/pseudogene)(UBE2NL)
Q5QNW6	H2B clustered histone 18(H2BC18)
Q5R3I4	tetratricopeptide repeat domain 38(TTC38)
Q5SSJ5	heterochromatin protein 1 binding protein 3(HP1BP3)
Q5SZK8	FRAS1 related extracellular matrix 2(FREM2)
Q5T1J5	coiled-coil-helix-coiled-coil-helix domain containing 2 pseudogene 9(CHCHD2P9)
Q5T4S7	ubiquitin protein ligase E3 component n-recognin 4(UBR4)
Q5TBC7	BCL2 like 15(BCL2L15)
Q5U651	Ras interacting protein 1(RASIP1)
Q5VT06	centrosomal protein 350(CEP350)
Q5VT66	mitochondrial amidoxime reducing component 1(MTARC1)
Q5VTE0	eukaryotic translation elongation factor 1 alpha 1 pseudogene 5(EEF1A1P5)
Q5XKE5	keratin 79(KRT79)
Q5XQN5	keratin 5(KRT5)

Q66LE6	protein phosphatase 2 regulatory subunit Bdelta(PPP2R2D)
Q6DD88	atlastin GTPase 3(ATL3)
Q6DKI2	galectin 9C(LGALS9C)
Q6EEV6	small ubiquitin like modifier 4(SUMO4)
Q6FI13	H2A clustered histone 18(H2AC18)
Q6FI13	H2A clustered histone 19(H2AC19)
Q6IFZ6	keratin 77(Krt77)
Q6IS14	eukaryotic translation initiation factor 5A like 1(EIF5AL1)
Q6NVY1	3-hydroxyisobutyryl-CoA hydrolase(HIBCH)
Q6NZI2	caveolae associated protein 1(CAVIN1)
Q6P4A8	phospholipase B domain containing 1(PLBD1)
Q6P587	fumarylacetoacetate hydrolase domain containing 1(FAHD1)
Q6P996	pyridoxal dependent decarboxylase domain containing 1(PDXDC1)
Q6PI48	aspartyl-tRNA synthetase 2, mitochondrial(DARS2)
Q6S8J3	POTE ankyrin domain family member E(POTEE)
Q6UWP2	dehydrogenase/reductase 11(DHRS11)
Q6UX06	olfactomedin 4(OLFM4)
Q6WRI0	immunoglobulin superfamily member 10(IGSF10)
Q6XQN6	nicotinate phosphoribosyltransferase(NAPRT)
Q6Y7W6	GRB10 interacting GYF protein 2(GIGYF2)
Q6YN16	hydroxysteroid dehydrogenase like 2(HSDL2)
Q71DI3	H3 clustered histone 14(H3C14)
Q71DI3	H3 clustered histone 15(H3C15)
Q71DI3	H3 clustered histone 13(H3C13)
Q71U36	tubulin alpha 1a(TUBA1A)
Q71UI9	H2A.Z variant histone 2(H2AZ2)
Q71UM5	ribosomal protein S27 like(RPS27L)
Q76L83	ASXL transcriptional regulator 2(ASXL2)

Q7KZF4	staphylococcal nuclease and tudor domain containing 1(SND1)
Q7L1Q6	basic leucine zipper and W2 domains 1(BZW1)
Q7L2H7	eukaryotic translation initiation factor 3 subunit M(EIF3M)
Q7L7L0	H2A clustered histone 25(H2AC25)
Q7LBR1	charged multivesicular body protein 1B(CHMP1B)
Q7Z3D6	D-glutamate cyclase(DGLUCY)
Q7Z406	myosin heavy chain 14(MYH14)
Q7Z4W1	dicarbonyl and L-xylulose reductase(DCXR)
Q7Z794	keratin 77(KRT77)
Q7Z7H5	transmembrane p24 trafficking protein 4(TMED4)
Q86SE5	RALY RNA binding protein like(RALYL)
Q86SE8	nucleophosmin/nucleoplasmin 2(NPM2)
Q86SF2	polypeptide N-acetylgalactosaminyltransferase 7(GALNT7)
Q86TX2	acyl-CoA thioesterase 1(ACOT1)
Q86U42	poly(A) binding protein nuclear 1(PABPN1)
Q86UP2	kinectin 1(KTN1)
Q86UX7	FERM domain containing kindlin 3(FERMT3)
Q86V81	Aly/REF export factor(ALYREF)
Q86VP6	cullin associated and neddylation dissociated 1(CAND1)
Q86WA8	lon peptidase 2, peroxisomal(LONP2)
Q86Y07	VRK serine/threonine kinase 2(VRK2)
Q86YZ3	hornerin(HRNR)
Q8BGZ7	keratin 75(Krt75)
Q8IUE6	H2A clustered histone 21(H2AC21)
Q8IV08	phospholipase D family member 3(PLD3)
Q8IVF4	dynein axonemal heavy chain 10(DNAH10)
Q8IXS8	hyccin PI4KA lipid kinase complex subunit 2(HYCC2)
Q8IZL8	proline, glutamate and leucine rich protein 1(PELP1)

Q8IZP2	ST13, Hsp70 interacting protein pseudogene 4(ST13P4)
Q8IZQ1	WD repeat and FYVE domain containing 3(WDFY3)
Q8N0X4	citramalyl-CoA lyase(CLYBL)
Q8N139	ATP binding cassette subfamily A member 6(ABCA6)
Q8N163	cell cycle and apoptosis regulator 2(CCAR2)
Q8N1G4	leucine rich repeat containing 47(LRRC47)
Q8N1N4	keratin 78(KRT78)
Q8N257	H2B clustered histone 26(H2BC26)
Q8N339	metallothionein 1M(MT1M)
Q8N3J5	protein phosphatase, Mg2+/Mn2+ dependent 1K(PPM1K)
Q8N3R3	T cell activation inhibitor, mitochondrial(TCAIM)
Q8N6T7	sirtuin 6(SIRT6)
Q8N8A2	ankyrin repeat domain 44(ANKRD44)
Q8NBJ7	sulfatase modifying factor 2(SUMF2)
Q8NBS9	thioredoxin domain containing 5(TXNDC5)
Q8NBX0	saccharopine dehydrogenase (putative)(SCCPDH)
Q8NC51	SERPINE1 mRNA binding protein 1(SERBP1)
Q8NE62	choline dehydrogenase(CHDH)
Q8NFF5	flavin adenine dinucleotide synthetase 1(FLAD1)
Q8NFI4	ST13, Hsp70 interacting protein pseudogene 5(ST13P5)
Q8NFU3	thiosulfate sulfurtransferase like domain containing 1(TSTD1)
Q8NFV4	abhydrolase domain containing 11(ABHD11)
Q8NFW8	cytidine monophosphate N-acetylneuraminic acid synthetase(CMAS)
Q8NHP1	aldo-keto reductase family 7 like (gene/pseudogene)(AKR7L)
Q8NHW5	ribosomal protein lateral stalk subunit P0 pseudogene 6(RPLP0P6)
Q8TAA3	proteasome 20S subunit alpha 8(PSMA8)
Q8TC12	retinol dehydrogenase 11(RDH11)
Q8TCD5	5', 3'-nucleotidase, cytosolic(NT5C)

Q8TD06	anterior gradient 3, protein disulphide isomerase family member(AGR3)
Q8WU39	marginal zone B and B1 cell specific protein(MZB1)
Q8WUD1	RAB2B, member RAS oncogene family(RAB2B)
Q8WUM4	programmed cell death 6 interacting protein(PDCD6IP)
Q8WWA0	intelectin 1(ITLN1)
Q8WXD9	CASK interacting protein 1(CASKIN1)
Q8WXF1	paraspeckle component 1(PSPC1)
Q8WXH0	spectrin repeat containing nuclear envelope protein 2(SYNE2)
Q92499	DEAD-box helicase 1(DDX1)
Q92506	hydroxysteroid 17-beta dehydrogenase 8(HSD17B8)
Q92597	N-myc downstream regulated 1(NDRG1)
Q92616	GCN1 activator of EIF2AK4(GCN1)
Q92628	KIAA0232(KIAA0232)
Q92688	acidic nuclear phosphoprotein 32 family member B(ANP32B)
Q92769	histone deacetylase 2(HDAC2)
Q92794	lysine acetyltransferase 6A(KAT6A)
Q92820	gamma-glutamyl hydrolase(GGH)
Q92841	DEAD-box helicase 17(DDX17)
Q92859	neogenin 1(NEO1)
Q92878	RAD50 double strand break repair protein(RAD50)
Q92896	golgi glycoprotein 1(GLG1)
Q92928	RAB1C, member RAS oncogene family pseudogene(RAB1C)
Q92945	KH-type splicing regulatory protein(KHSRP)
Q92973	transportin 1(TNPO1)
Q93052	LIM domain containing preferred translocation partner in lipoma(LPP)
Q93077	H2A clustered histone 6(H2AC6)
Q93079	H2B clustered histone 9(H2BC9)
Q93084	ATPase sarcoplasmic/endoplasmic reticulum Ca2+ transporting 3(ATP2A3)

Q969H8	myeloid derived growth factor(MYDGF)
Q969Q0	ribosomal protein L36a like(RPL36AL)
Q969Z3	mitochondrial amidoxime reducing component 2(MTARC2)
Q96AB3	isochorismatase domain containing 2(ISOC2)
Q96AE4	far upstream element binding protein 1(FUBP1)
Q96BQ1	FAM3 metabolism regulating signaling molecule D(FAM3D)
Q96C23	galactose mutarotase(GALM)
Q96CM8	acyl-CoA synthetase family member 2(ACSF2)
Q96CX2	potassium channel tetramerization domain containing 12(KCTD12)
Q96G03	phosphoglucomutase 2(PGM2)
Q96HE7	endoplasmic reticulum oxidoreductase 1 alpha(ERO1A)
Q96HN2	adenosylhomocysteinase like 2(AHCYL2)
Q96HS1	PGAM family member 5, mitochondrial serine/threonine protein phosphatase(PGAM5)
Q96I99	succinate-CoA ligase GDP-forming subunit beta(SUCLG2)
Q96IU4	abhydrolase domain containing 14B(ABHD14B)
Q96JN2	coiled-coil domain containing 136(CCDC136)
Q96JQ0	dachsous cadherin-related 1(DCHS1)
Q96JY0	maelstrom spermatogenic transposon silencer(MAEL)
Q96KJ4	mesothelin like(MSLNL)
Q96KK5	H2A clustered histone 12(H2AC12)
Q96KP4	carnosine dipeptidase 2(CNDP2)
Q96L21	ribosomal protein L10 like(RPL10L)
Q96L93	kinesin family member 16B(KIF16B)
Q96LJ7	dehydrogenase/reductase 1(DHRS1)
Q96M69	leucine rich repeats and guanylate kinase domain containing(LRGUK)
Q96N66	membrane bound O-acyltransferase domain containing 7(MBOAT7)
Q96P70	importin 9(IPO9)
Q96PK6	RNA binding motif protein 14(RBM14)

Q96QK1	VPS35 retromer complex component(VPS35)
Q96QS3	aristaless related homeobox(ARX)
Q96QV6	H2A clustered histone 1(H2AC1)
Q96RQ3	methylcrotonyl-CoA carboxylase subunit 1(MCCC1)
Q96RY5	cramped chromatin regulator homolog 1(CRAMP1)
Q96S21	RAB40C, member RAS oncogene family(RAB40C)
Q96TA1	niban apoptosis regulator 2(NIBAN2)
Q99436	proteasome 20S subunit beta 7(PSMB7)
Q99460	proteasome 26S subunit, non-ATPase 1(PSMD1)
Q99497	Parkinsonism associated deglycase(PARK7)
Q99536	vesicle amine transport 1(VAT1)
Q99598	translin associated factor X(TSNAX)
Q99613	eukaryotic translation initiation factor 3 subunit C(EIF3C)
Q99623	prohibitin 2(PHB2)
Q99714	hydroxysteroid 17-beta dehydrogenase 10(HSD17B10)
Q99729	heterogeneous nuclear ribonucleoprotein A/B(HNRNPAB)
Q99733	nucleosome assembly protein 1 like 4(NAP1L4)
Q99735	microsomal glutathione S-transferase 2(MGST2)
Q99757	thioredoxin 2(TXN2)
Q99795	glycoprotein A33(GPA33)
Q99798	aconitase 2(ACO2)
Q99829	copine 1(CPNE1)
Q99832	chaperonin containing TCP1 subunit 7(CCT7)
Q99877	H2B clustered histone 15(H2BC15)
Q99879	H2B clustered histone 14(H2BC14)
Q99880	H2B clustered histone 13(H2BC13)
Q99933	BAG cochaperone 1(BAG1)
Q9BPW8	nipsnap homolog 1(NIPSNAP1)

Q9BPX5	actin related protein 2/3 complex subunit 5 like(ARPC5L)
Q9BQ69	mono-ADP ribosylhydrolase 1(MACROD1)
Q9BQE3	tubulin alpha 1c(TUBA1C)
Q9BR76	coronin 1B(CORO1B)
Q9BRA2	thioredoxin domain containing 17(TXNDC17)
Q9BRL6	serine and arginine rich splicing factor 8(SRSF8)
Q9BRP8	PYM homolog 1, exon junction complex associated factor(PYM1)
Q9BS26	endoplasmic reticulum protein 44(ERP44)
Q9BSH4	translational activator of cytochrome c oxidase I(TACO1)
Q9BSH5	haloacid dehalogenase like hydrolase domain containing 3(HDHD3)
Q9BSJ8	extended synaptotagmin 1(ESYT1)
Q9BTM1	H2A.J histone(H2AJ)
Q9BUF5	tubulin beta 6 class V(TUBB6)
Q9BUJ2	heterogeneous nuclear ribonucleoprotein U like 1(HNRNPUL1)
Q9BUT1	3-hydroxybutyrate dehydrogenase 2(BDH2)
Q9BV35	solute carrier family 25 member 23(SLC25A23)
Q9BVA1	tubulin beta 2B class IIb(TUBB2B)
Q9BVC6	transmembrane protein 109(TMEM109)
Q9BVK6	transmembrane p24 trafficking protein 9(TMED9)
Q9BX68	histidine triad nucleotide binding protein 2(HINT2)
Q9BXW7	haloacid dehalogenase like hydrolase domain containing 5(HDHD5)
Q9BYX7	POTE ankyrin domain family member K, pseudogene(POTEKP)
Q9BZJ3	tryptase delta 1(TPSD1)
Q9BZZ5	apoptosis inhibitor 5(API5)
Q9C0B6	BMP/retinoic acid inducible neural specific 2(BRINP2)
Q9GZT3	SRA stem-loop interacting RNA binding protein(SLIRP)
Q9GZV4	eukaryotic translation initiation factor 5A2(EIF5A2)
Q9H008	phospholysine phosphohistidine inorganic pyrophosphate phosphatase(LHPP)

Q9H0C2	solute carrier family 25 member 31(SLC25A31)
Q9H0U4	RAB1B, member RAS oncogene family(RAB1B)
Q9H1E3	nuclear casein kinase and cyclin dependent kinase substrate 1(NUCKS1)
Q9H223	EH domain containing 4(EHD4)
Q9H299	SH3 domain binding glutamate rich protein like 3(SH3BGRL3)
Q9H2G2	STE20 like kinase(SLK)
Q9H2U2	inorganic pyrophosphatase 2(PPA2)
Q9H361	poly(A) binding protein cytoplasmic 3(PABPC3)
Q9H3P7	acyl-CoA binding domain containing 3(ACBD3)
Q9H444	charged multivesicular body protein 4B(CHMP4B)
Q9H4A4	arginyl aminopeptidase(RNPEP)
Q9H4B7	tubulin beta 1 class VI(TUBB1)
Q9H4G4	GLI pathogenesis related 2(GLIPR2)
Q9H4M9	EH domain containing 1(EHD1)
Q9H6E5	terminal uridylyl transferase 1, U6 snRNA-specific(TUT1)
Q9H7P9	pleckstrin homology and RhoGEF domain containing G2(PLEKHG2)
Q9H7Z3	NRDE-2, necessary for RNA interference, domain containing(NRDE2)
Q9H8H3	methyltransferase like 7A(METTL7A)
Q9H9B4	sideroflexin 1(SFXN1)
Q9HAV0	G protein subunit beta 4(GNB4)
Q9HAW7	UDP glucuronosyltransferase family 1 member A7(UGT1A7)
Q9HAW8	UDP glucuronosyltransferase family 1 member A10(UGT1A10)
Q9HAW9	UDP glucuronosyltransferase family 1 member A8(UGT1A8)
Q9HB71	calcyclin binding protein(CACYBP)
Q9HC35	EMAP like 4(EML4)
Q9HC38	glyoxalase domain containing 4(GLOD4)
Q9HC84	mucin 5B, oligomeric mucus/gel-forming(MUC5B)
Q9HCC0	methylcrotonyl-CoA carboxylase subunit 2(MCCC2)

Q9HCJ0	trinucleotide repeat containing adaptor 6C(TNRC6C)
Q9HCY8	S100 calcium binding protein A14(S100A14)
Q9HDC9	adipocyte plasma membrane associated protein(APMAP)
Q9NPF4	O-sialoglycoprotein endopeptidase(OSGEP)
Q9NQR4	nitrilase family member 2(NIT2)
Q9NR28	diablo IAP-binding mitochondrial protein(DIABLO)
Q9NR31	secretion associated Ras related GTPase 1A(SAR1A)
Q9NR45	N-acetylneuraminate synthase(NANS)
Q9NRV9	heme binding protein 1(HEBP1)
Q9NRX4	phosphohistidine phosphatase 1(PHPT1)
Q9NS69	translocase of outer mitochondrial membrane 22(TOMM22)
Q9NSD9	phenylalanyl-tRNA synthetase subunit beta(FARSB)
Q9NSE4	isoleucyl-tRNA synthetase 2, mitochondrial(IARS2)
Q9NSK0	kinesin light chain 4(KLC4)
Q9NTK5	Obg like ATPase 1(OLA1)
Q9NUJ1	abhydrolase domain containing 10, depalmitoylase(ABHD10)
Q9NUP9	lin-7 homolog C, crumbs cell polarity complex component(LIN7C)
Q9NUQ9	CYFIP related Rac1 interactor B(CYRIB)
Q9NUR3	transmembrane protein 74B(TMEM74B)
Q9NVA2	septin 11(SEPTIN11)
Q9NVJ2	ADP ribosylation factor like GTPase 8B(ARL8B)
Q9NX63	coiled-coil-helix-coiled-coil-helix domain containing 3(CHCHD3)
Q9NXZ2	DEAD-box helicase 43(DDX43)
Q9NY33	dipeptidyl peptidase 3(DPP3)
Q9NYC9	dynein axonemal heavy chain 9(DNAH9)
Q9NYU2	UDP-glucose glycoprotein glucosyltransferase 1(UGGT1)
Q9NYV4	cyclin dependent kinase 12(CDK12)
Q9NZ08	endoplasmic reticulum aminopeptidase 1(ERAP1)

Q9NZ45	CDGSH iron sulfur domain 1(CISD1)
Q9NZB2	family with sequence similarity 120A(FAM120A)
Q9NZL9	methionine adenosyltransferase 2B(MAT2B)
Q9NZN3	EH domain containing 3(EHD3)
Q9NZN4	EH domain containing 2(EHD2)
Q9NZN5	Rho guanine nucleotide exchange factor 12(ARHGEF12)
Q9P1U1	actin related protein 3B(ACTR3B)
Q9P225	dynein axonemal heavy chain 2(DNAH2)
Q9P241	ATPase phospholipid transporting 10D (putative)(ATP10D)
Q9P2E9	ribosome binding protein 1(RRBP1)
Q9P2K8	eukaryotic translation initiation factor 2 alpha kinase 4(EIF2AK4)
Q9P2R7	succinate-CoA ligase ADP-forming subunit beta(SUCLA2)
Q9P2T1	guanosine monophosphate reductase 2(GMPR2)
Q9UBE0	SUMO1 activating enzyme subunit 1(SAE1)
Q9UBF2	COPI coat complex subunit gamma 2(COPG2)
Q9UBI6	G protein subunit gamma 12(GNG12)
Q9UBQ0	VPS29 retromer complex component(VPS29)
Q9UBQ7	glyoxylate and hydroxypyruvate reductase(GRHPR)
Q9UBR2	cathepsin Z(CTSZ)
Q9UBS4	DnaJ heat shock protein family (Hsp40) member B11(DNAJB11)
Q9UBV8	penta-EF-hand domain containing 1(PEF1)
Q9UDY2	tight junction protein 2(TJP2)
Q9UFH2	dynein axonemal heavy chain 17(DNAH17)
Q9UGM3	deleted in malignant brain tumors 1(DMBT1)
Q9UHB6	LIM domain and actin binding 1(LIMA1)
Q9UHD8	septin 9(SEPTIN9)
Q9UII2	ATP synthase inhibitory factor subunit 1(ATP5IF1)
Q9UIJ7	adenylate kinase 3(AK3)

Q9UJ41	RAB guanine nucleotide exchange factor 1(RABGEF1)
Q9UJS0	solute carrier family 25 member 13(SLC25A13)
Q9UJU6	drebrin like(DBNL)
Q9UJX3	anaphase promoting complex subunit 7(ANAPC7)
Q9UJZ1	stomatin like 2(STOML2)
Q9UKM9	RALY heterogeneous nuclear ribonucleoprotein(RALY)
Q9UL46	proteasome activator subunit 2(PSME2)
Q9UL63	muskelin 1(MKLN1)
Q9ULA0	aspartyl aminopeptidase(DNPEP)
Q9ULC5	acyl-CoA synthetase long chain family member 5(ACSL5)
Q9ULV4	coronin 1C(CORO1C)
Q9ULX5	ring finger protein 112(RNF112)
Q9ULZ3	PYD and CARD domain containing(PYCARD)
Q9UNH7	sorting nexin 6(SNX6)
Q9UNL2	signal sequence receptor subunit 3(SSR3)
Q9UNX3	ribosomal protein L26 like 1(RPL26L1)
Q9UNZ2	NSFL1 cofactor(NSFL1C)
Q9UPY3	dicer 1, ribonuclease III(DICER1)
Q9UQ80	proliferation-associated 2G4(PA2G4)
Q9UQE7	structural maintenance of chromosomes 3(SMC3)
Q9Y262	eukaryotic translation initiation factor 3 subunit L(EIF3L)
Q9Y265	RuvB like AAA ATPase 1(RUVBL1)
Q9Y277	voltage dependent anion channel 3(VDAC3)
Q9Y285	phenylalanyl-tRNA synthetase subunit alpha(FARSA)
Q9Y2A7	NCK associated protein 1(NCKAP1)
Q9Y2B0	canopy FGF signaling regulator 2(CNPY2)
Q9Y2C9	toll like receptor 6(TLR6)
Q9Y2J8	peptidyl arginine deiminase 2(PADI2)

Q9Y2Q3	glutathione S-transferase kappa 1(GSTK1)
Q9Y2Y8	proteoglycan 3, pro eosinophil major basic protein 2(PRG3)
Q9Y315	deoxyribose-phosphate aldolase(DERA)
Q9Y365	StAR related lipid transfer domain containing 10(STARD10)
Q9Y3B8	RNA exonuclease 2(REXO2)
Q9Y3D6	fission, mitochondrial 1(FIS1)
Q9Y3F4	serine/threonine kinase receptor associated protein(STRAP)
Q9Y3U8	ribosomal protein L36(RPL36)
Q9Y3Z3	SAM and HD domain containing deoxynucleoside triphosphate triphosphohydrolase 1(SAMHD1)
Q9Y490	talin 1(TLN1)
Q9Y4F4	TOG array regulator of axonemal microtubules 1(TOGARAM1)
Q9Y4L1	hypoxia up-regulated 1(HYOU1)
Q9Y4P3	transducin beta like 2(TBL2)
Q9Y4W6	AFG3 like matrix AAA peptidase subunit 2(AFG3L2)
Q9Y512	SAMM50 sorting and assembly machinery component(SAMM50)
Q9Y5L4	translocase of inner mitochondrial membrane 13(TIMM13)
Q9Y5M8	SRP receptor subunit beta(SRPRB)
Q9Y5P6	GDP-mannose pyrophosphorylase B(GMPPB)
Q9Y5S9	RNA binding motif protein 8A(RBM8A)
Q9Y618	nuclear receptor corepressor 2(NCOR2)
Q9Y678	COPI coat complex subunit gamma 1(COPG1)
Q9Y6B6	secretion associated Ras related GTPase 1B(SAR1B)
Q9Y6E2	basic leucine zipper and W2 domains 2(BZW2)
Q9Y6H1	coiled-coil-helix-coiled-coil-helix domain containing 2(CHCHD2)
Q9Y6N5	sulfide quinone oxidoreductase(SQOR)
Q9Y6R7	Fc gamma binding protein(FCGBP)
Q9Y6W5	WASP family member 2(WASF2)
